# Supplementary material for: IsoGraph resolves coordinated transcript choice and gene abundance in the aging human brain
Source: bioRxiv. 2026 Oct 11:2026.10.04.756439. Preprint. [Version 1] doi: 10.64898/2026.10.04.756439 (PMC13692093; doi:10.64898/2026.10.04.756439)
Supplement: Supplementary file 1 [file NIHPP2026.10.04.756439V1-supplement-1.pdf]

bioRxiv preprint doi: https://doi.org/10.64898/2026.10.04.756439; this version posted October 11, 2026. The copyright holder for this preprint (which was not certified by peer review) is the author/funder, who has granted bioRxiv a license to display the preprint in perpetuity. It is made available under a CC-BY 4.0 International license.

Supplementary Information: IsoGraph resolves coordinated transcript choice and gene abundance in the aging human brain

Authors

- **Alexis Bennett**
  0000-0002-5384-7657
  Department of Psychiatry and Behavioral Sciences, Feinberg School of Medicine, Northwestern University

- **Elisa Kain Johnson**
  0009-0009-2187-3728
  Weinberg College of Arts and Sciences, Northwestern University; Department of Psychiatry and Behavioral Sciences, Feinberg School of Medicine, Northwestern University

- **Kynon J M Benjamin** ✉
  0000-0003-2016-4646
  Stephen M. Stahl Center for Psychiatric Neuroscience, Feinberg School of Medicine, Northwestern University; Department of Psychiatry and Behavioral Sciences, Feinberg School of Medicine, Northwestern University · Funded by NIH Grant R00MD016964; Alzheimer's Association 25AARG-1413315; Brain & Behavior Research Foundation Young Investigator Grant; NSF ACCESS allocation BIO260136

✉ — Correspondence possible by email to Kynon J M Benjamin <kynon.benjamin@northwestern.edu>.

bioRxiv preprint doi: https://doi.org/10.64898/2026.10.04.756439; this version posted October 11, 2026. The copyright holder for this preprint (which was not certified by peer review) is the author/funder, who has granted bioRxiv a license to display the preprint in perpetuity. It is made available under a CC-BY 4.0 International license.

Supplementary Figures

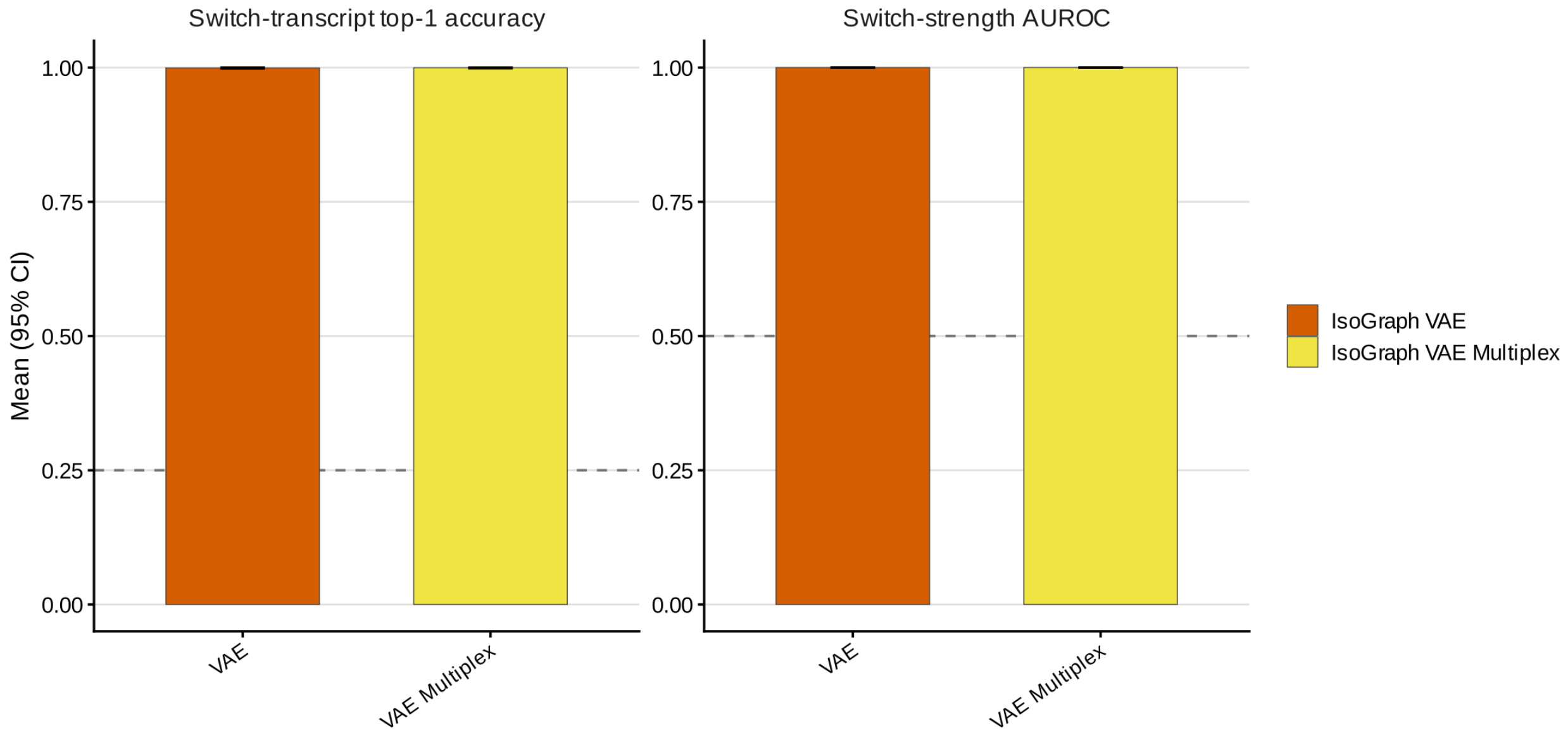

Fig. S1: Module interpretation recovers planted switch transcripts and switching genes. Left, switch-transcript top-1 accuracy: the fraction of switching genes whose highest-loading transcript is the planted switch transcript, evaluated in multi-isoform simulations (four transcripts per gene; dashed line, chance = 0.25). Right, switch-strength area under the receiver operating characteristic curve (AUROC) for separating switching from non-switching genes within modules (dashed line, chance = 0.5). Bars show means across simulations for IsoGraph VAE (variational autoencoder; switch-only) and IsoGraph VAE Multiplex; error bars indicate 95% bootstrap confidence intervals.

bioRxiv preprint doi: https://doi.org/10.64898/2026.10.04.756439; this version posted October 11, 2026. The copyright holder for this preprint (which was not certified by peer review) is the author/funder, who has granted bioRxiv a license to display the preprint in perpetuity. It is made available under a CC-BY 4.0 International license.

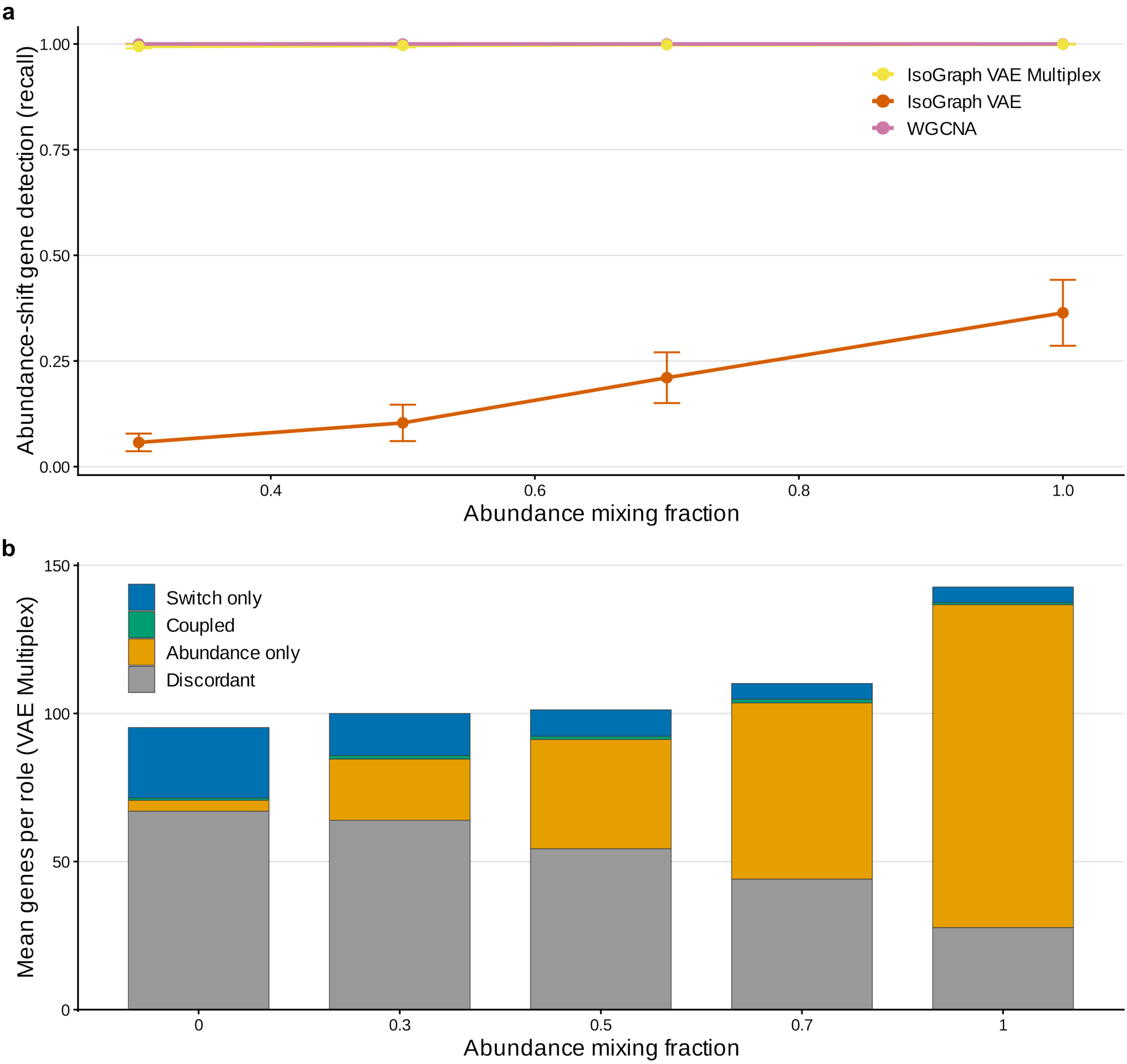

Fig. S2: Mixed switch–abundance simulations: abundance-shift detection and channel-specific roles. Simulations mixed planted isoform switching with abundance shifts at increasing abundance mixing fractions. **a.** Recall of genes carrying a planted abundance shift for IsoGraph VAE (variational autoencoder), IsoGraph VAE Multiplex, and weighted gene co-expression network analysis (WGCNA). Points show means across simulation seeds and error bars indicate 95% confidence intervals. **b.** Mean number of genes assigned to each channel-specific role by IsoGraph VAE Multiplex: switch only, coupled (switch and abundance), abundance only, and discordant.

bioRxiv preprint doi: https://doi.org/10.64898/2026.10.04.756439; this version posted October 11, 2026. The copyright holder for this preprint (which was not certified by peer review) is the author/funder, who has granted bioRxiv a license to display the preprint in perpetuity. It is made available under a CC-BY 4.0 International license.

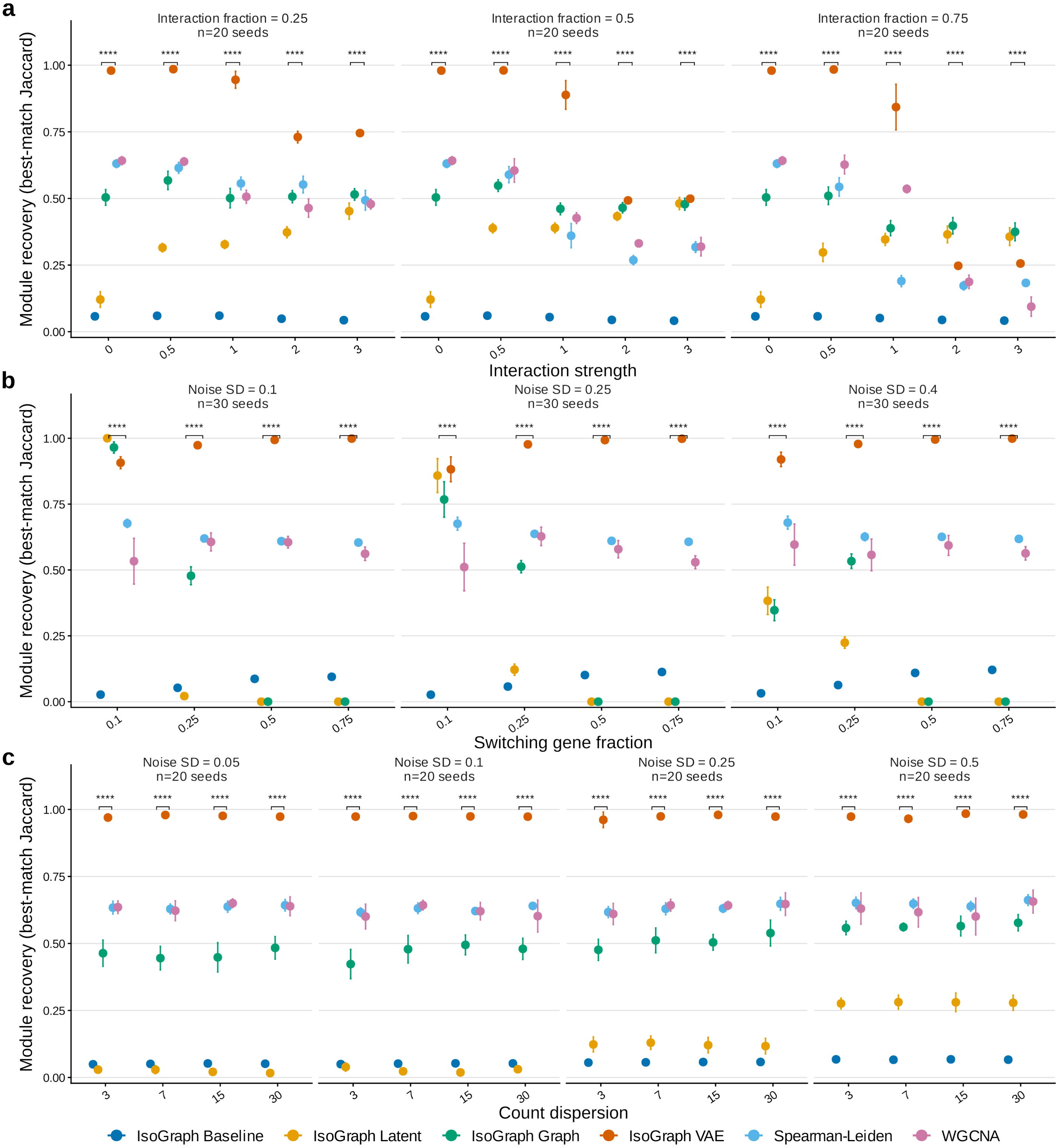

Fig. S3: Module recovery under feature interactions, noise, and count dispersion. a. Recovery as a function of interaction strength between the switch and abundance features, at interaction fractions of 0.25, 0.5 and 0.75 (20 seeds per setting). **b.** Recovery as a function of the fraction of switching genes at three noise levels (30 seeds per setting). **c.** Recovery as a function of count dispersion at four noise levels (20 seeds per setting). Module recovery is the best-match Jaccard similarity between each planted module and its closest inferred module (1 = perfect recovery). Points show means across simulation seeds and error bars indicate 95% confidence intervals. Asterisks mark two-sided Wilcoxon rank-sum comparisons of IsoGraph VAE (variational autoencoder) with weighted gene co-expression network analysis (WGCNA) at each parameter value, Benjamini–Hochberg-adjusted within each panel (**** adjusted P [P_{adj}] < 10^{-4}); these per-panel comparisons are for visual guidance and are separate from the full-family tests in Table S1.

bioRxiv preprint doi: https://doi.org/10.64898/2026.10.04.756439; this version posted October 11, 2026. The copyright holder for this preprint (which was not certified by peer review) is the author/funder, who has granted bioRxiv a license to display the preprint in perpetuity. It is made available under a CC-BY 4.0 International license.

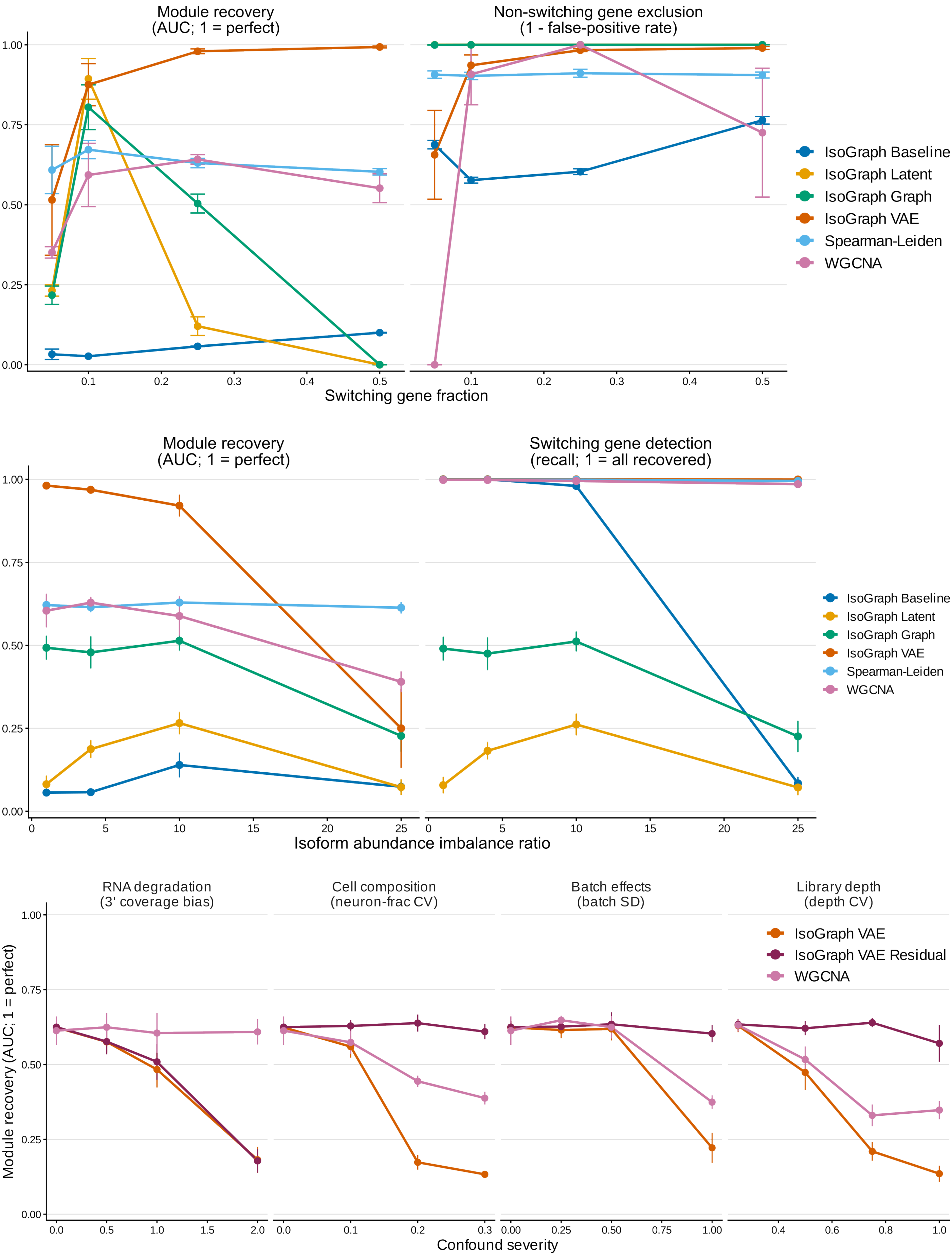

Fig. S6: Robustness to simulated technical and compositional confounders. Module recovery as the severity of four confounders increased: RNA degradation (3′ coverage bias), cell composition (coefficient of variation of the neuronal fraction), batch effects (batch standard deviation), and library depth (coefficient of variation of depth). IsoGraph VAE (variational autoencoder), its residualized implementation (IsoGraph VAE Residual), and weighted gene co-expression network analysis (WGCNA) are shown. Module recovery is the best-match Jaccard similarity between each

bioRxiv preprint doi: https://doi.org/10.64898/2026.10.04.756439; this version posted October 11, 2026. The copyright holder for this preprint (which was not certified by peer review) is the author/funder, who has granted bioRxiv a license to display the preprint in perpetuity. It is made available under a CC-BY 4.0 International license.

planted module and its closest inferred module (1 = perfect recovery). Points show means across simulation seeds and error bars indicate 95% confidence intervals.

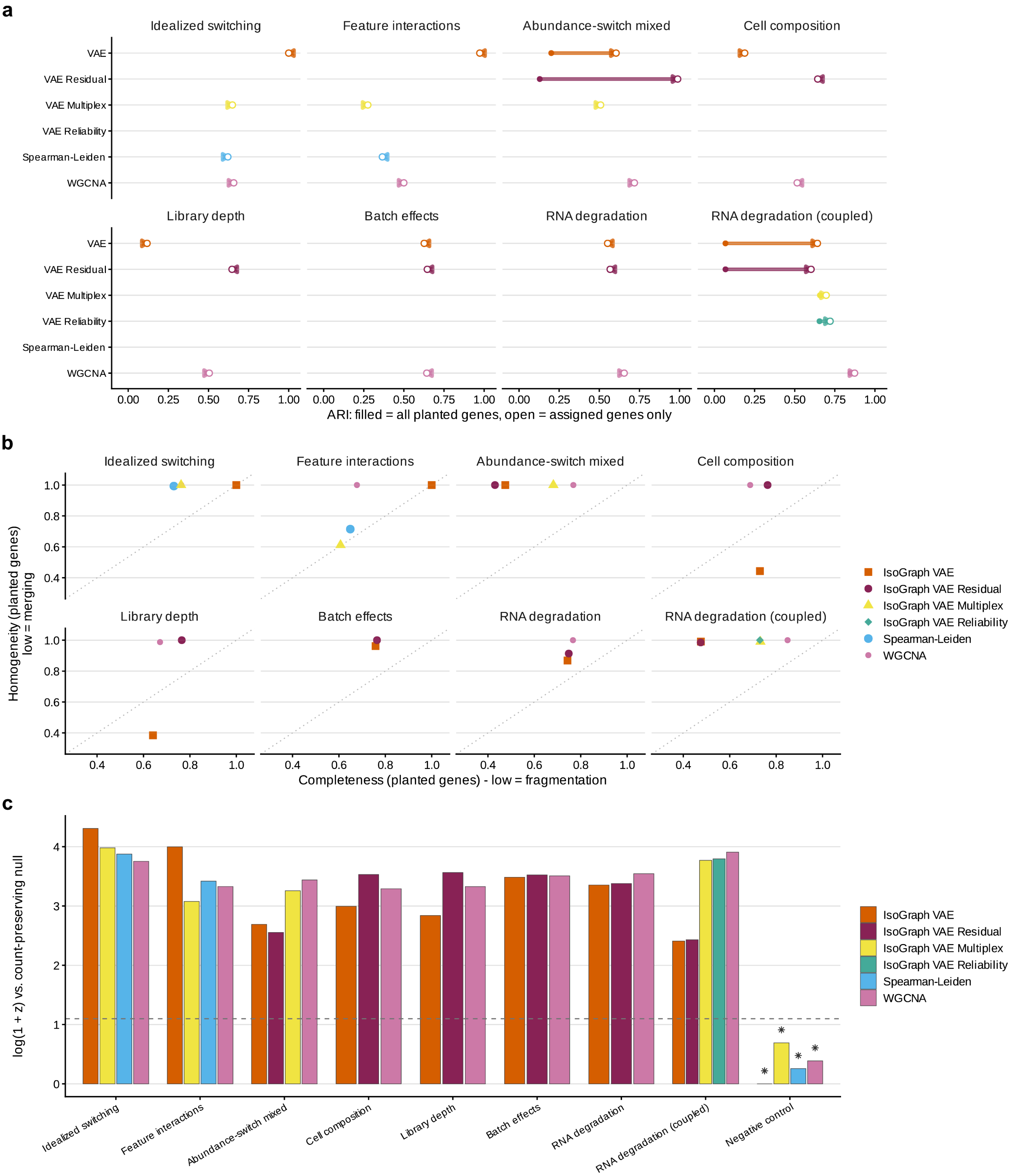

Fig. S7: Partition diagnostics complement best-match module recovery. a. Adjusted Rand index (ARI) against the planted partition, computed over all planted genes (filled) or only genes assigned to a module (open). **b.** Homogeneity versus completeness for planted genes; low homogeneity indicates merging of planted modules, and low completeness indicates fragmentation. **c.** Module recovery relative to a permutation null that preserves the number of inferred modules, shown as log(1 + z), where z is the observed recovery minus the null mean, divided by the null standard deviation. The dashed line marks $z = 2$; asterisks mark a median permutation $P > 0.05$, that is, recovery fully explained by module-count structure.

bioRxiv preprint doi: https://doi.org/10.64898/2026.10.04.756439; this version posted October 11, 2026. The copyright holder for this preprint (which was not certified by peer review) is the author/funder, who has granted bioRxiv a license to display the preprint in perpetuity. It is made available under a CC-BY 4.0 International license.

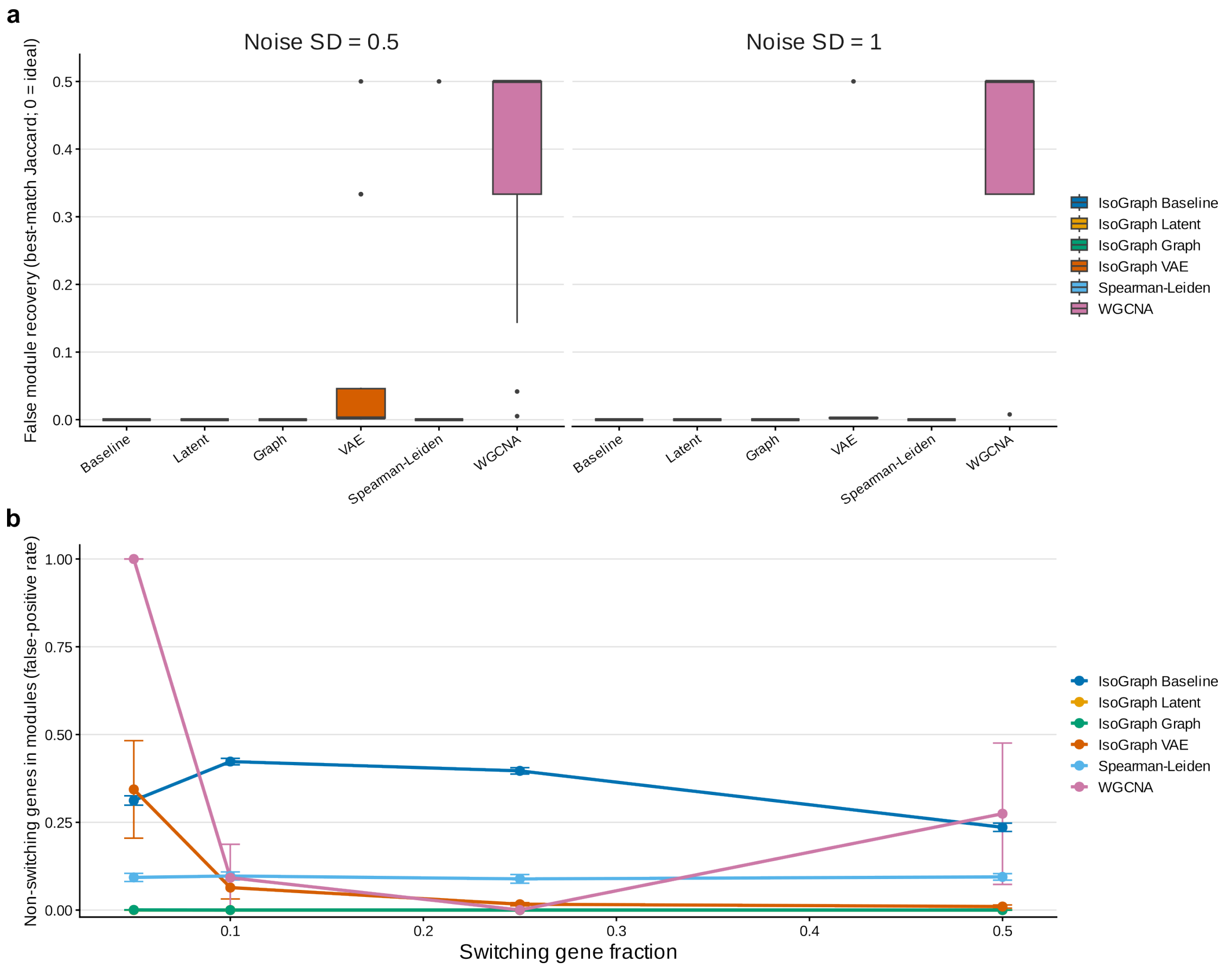

Fig. S8: Specificity under null and non-switching settings. a. False module recovery in pure-noise negative-control simulations at two noise levels (0 = ideal). **b.** Fraction of non-switching genes assigned to modules (false-positive rate) as a function of the fraction of switching genes. Boxplots in **a** summarize simulation seeds; points in **b** show means across seeds, with error bars indicating 95% confidence intervals.

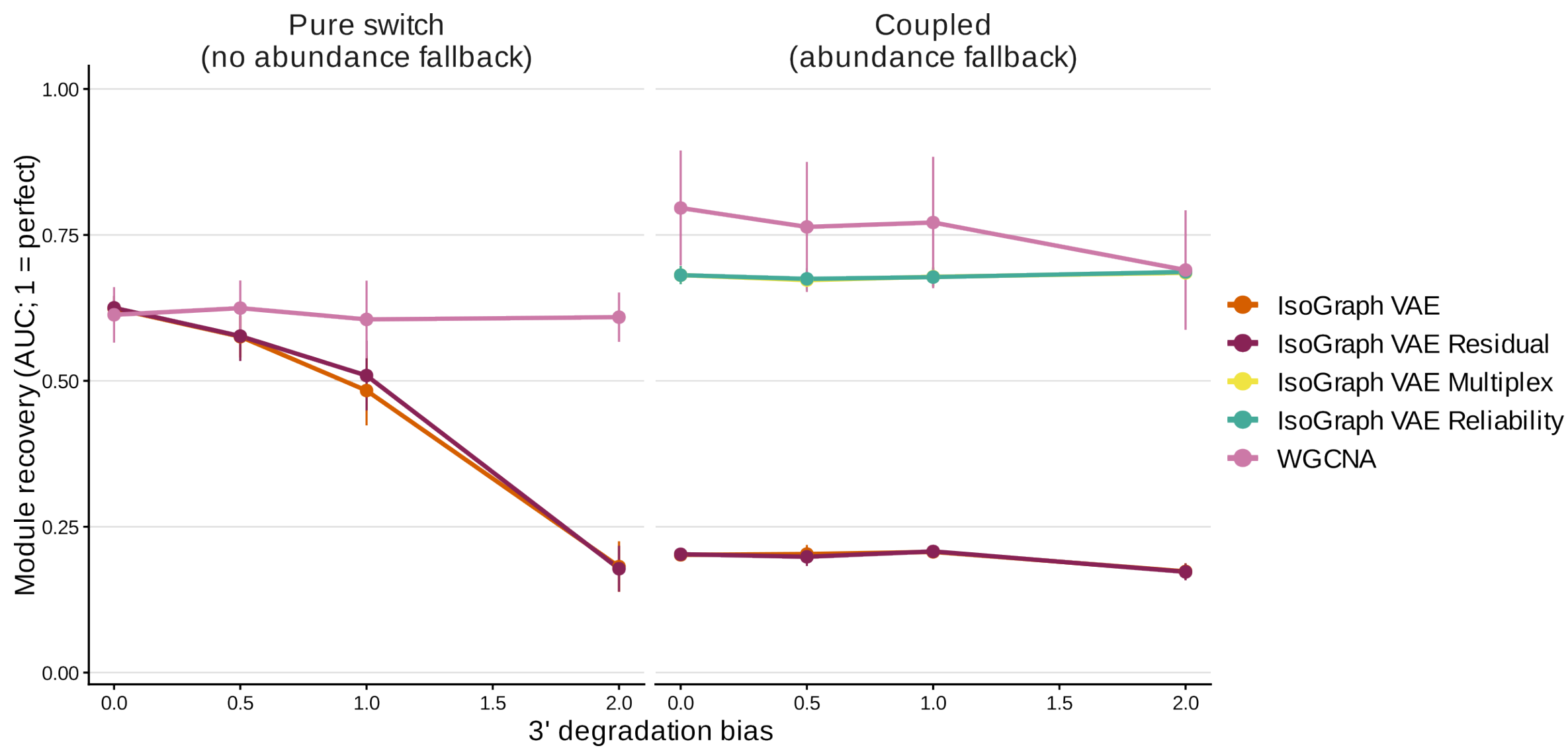

Fig. S9: Multiplex integration provides an abundance-based fallback when RNA degradation obscures isoform-switching structure. Module recovery was evaluated across increasing 3′-degradation bias under two regulatory architectures. In the pure-switch setting, planted modules were encoded only through coordinated isoform switching and therefore lacked an abundance signal that could support recovery. In the coupled setting, switching and gene-abundance signals were concordantly embedded in the same modules, permitting abundance-informed recovery. Performance was quantified by the mean maximum Jaccard similarity to planted modules, with 1 indicating perfect recovery. IsoGraph VAE (variational autoencoder) and its residualized implementation were evaluated in both settings and compared with weighted gene co-expression network analysis (WGCNA); the coupled setting additionally evaluated IsoGraph VAE Multiplex and reliability-weighted IsoGraph VAE. Points show mean performance across independent simulation seeds, error bars indicate 95% confidence intervals, and lines connect results obtained with the same method.

bioRxiv preprint doi: https://doi.org/10.64898/2026.10.04.756439; this version posted October 11, 2026. The copyright holder for this preprint (which was not certified by peer review) is the author/funder, who has granted bioRxiv a license to display the preprint in perpetuity. It is made available under a CC-BY 4.0 International license.

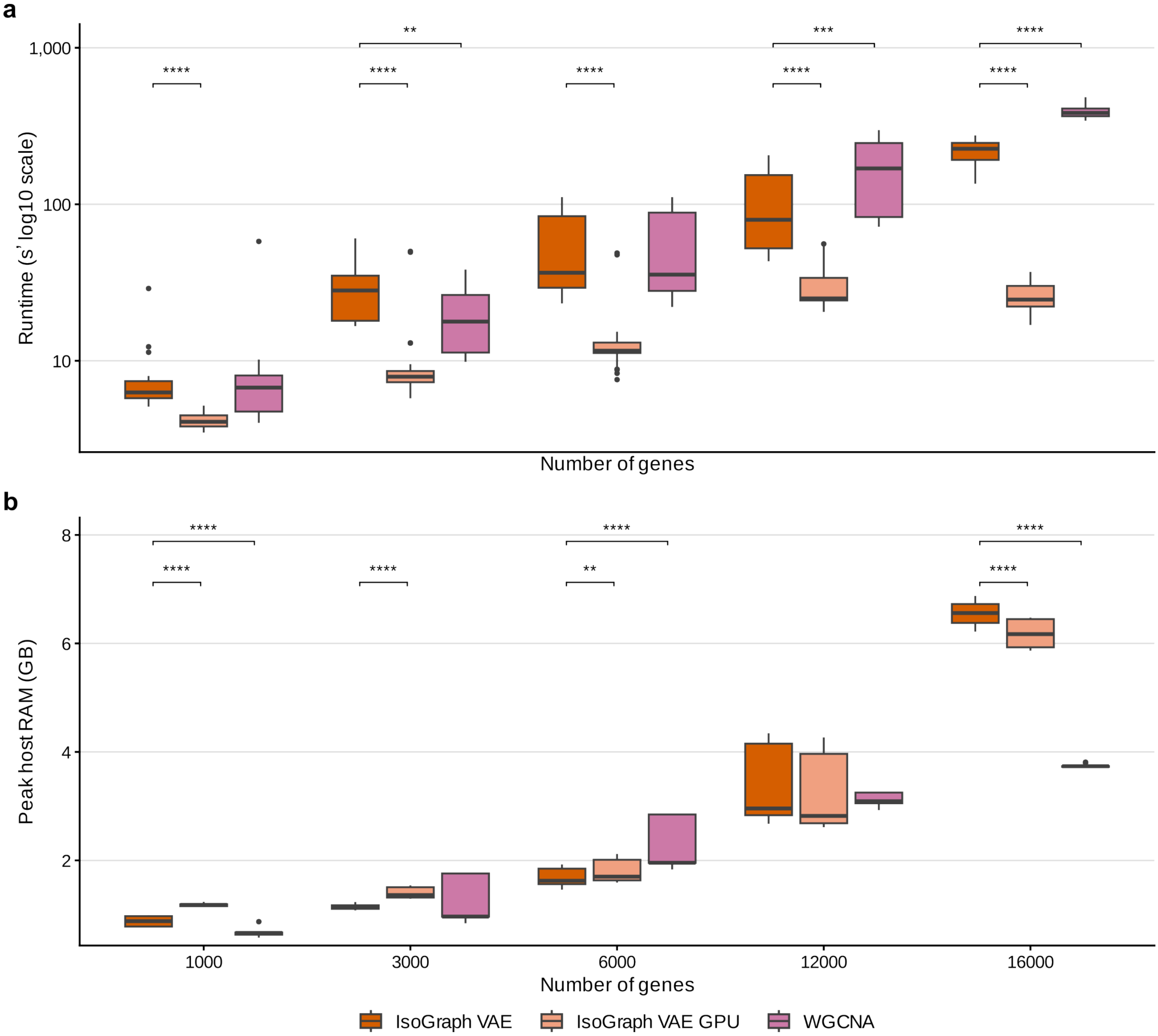

Fig. S10: Computational scaling. a. Runtime (seconds, $\log_{10}$ scale) and **b.** peak host memory (GB) as a function of the number of genes for IsoGraph VAE (variational autoencoder) on a central processing unit (CPU), IsoGraph VAE on a graphics processing unit (GPU), and weighted gene co-expression network analysis (WGCNA). Boxplots summarize replicate runs. Asterisks mark two-sided Wilcoxon rank-sum comparisons with IsoGraph VAE (CPU) at each gene count, Benjamini–Hochberg-adjusted within each panel (** adjusted P [P_{adj}] < 0.01; *** P_{adj} < 0.001; **** $P_{adj} < 10^{-4}$). Summary statistics are given in Table S2.

bioRxiv preprint doi: https://doi.org/10.64898/2026.10.04.756439; this version posted October 11, 2026. The copyright holder for this preprint (which was not certified by peer review) is the author/funder, who has granted bioRxiv a license to display the preprint in perpetuity. It is made available under a CC-BY 4.0 International license.

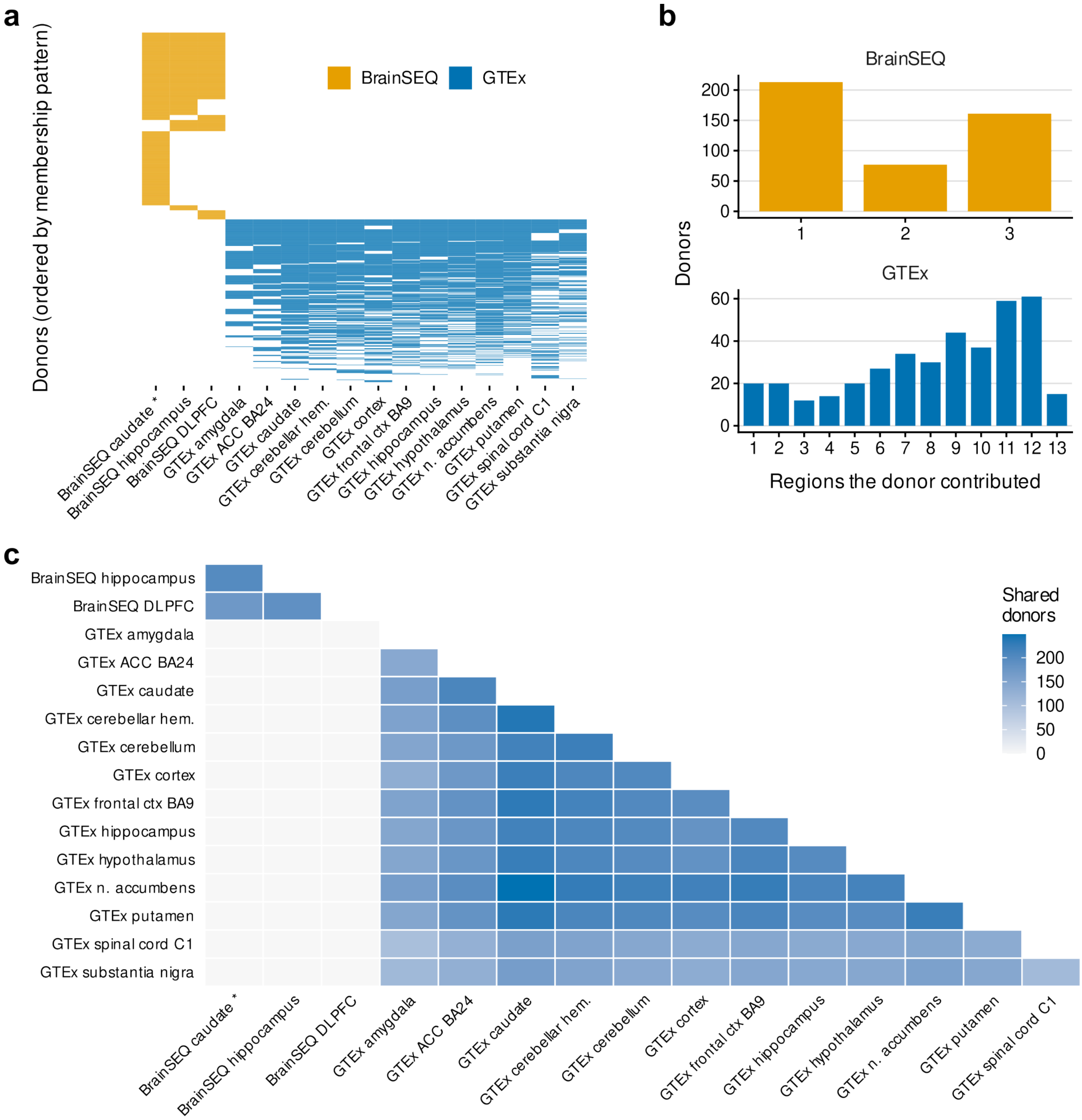

Fig. S11: Donor sharing across the 16 brain regions and 17 analyses. a. Donor-by-region incidence. Each row is a donor and each column a region; a filled cell indicates that the donor contributed tissue to that region. Donors are ordered by the set of regions they contributed, so shared-donor blocks are contiguous. Asterisks mark a region analyzed more than once. **b.** Number of regions each donor contributed, by cohort. **c.** Shared-donor counts for every pair of regions; blank cells indicate no shared donors. The 16 regions comprise three BrainSEQ and 13 Genotype-Tissue Expression (GTEx) regions, and are covered by 17 analyses because BrainSEQ caudate is analyzed twice: once for aging over its 238 control samples and once for schizophrenia over those same 238 control samples together with 152 samples from donors with schizophrenia. The 4,322 sample-analysis rows therefore correspond to 4,084 distinct tissue samples from 844 donors. Donor identifiers (BrNum for BrainSEQ, SUBJID for GTEx) are shared across regions within a cohort and are never matched across cohorts, whereas the sample identifier (RNum for BrainSEQ) is specific to a donor and region. The median GTEx donor contributed 9 of the 13 GTEx regions and the median BrainSEQ donor 2 of the 3 BrainSEQ regions, so agreement between regions within a cohort is re-measurement of a shared donor pool, whereas BrainSEQ-versus-GTEx comparisons carry independent donors.

bioRxiv preprint doi: https://doi.org/10.64898/2026.10.04.756439; this version posted October 11, 2026. The copyright holder for this preprint (which was not certified by peer review) is the author/funder, who has granted bioRxiv a license to display the preprint in perpetuity. It is made available under a CC-BY 4.0 International license.

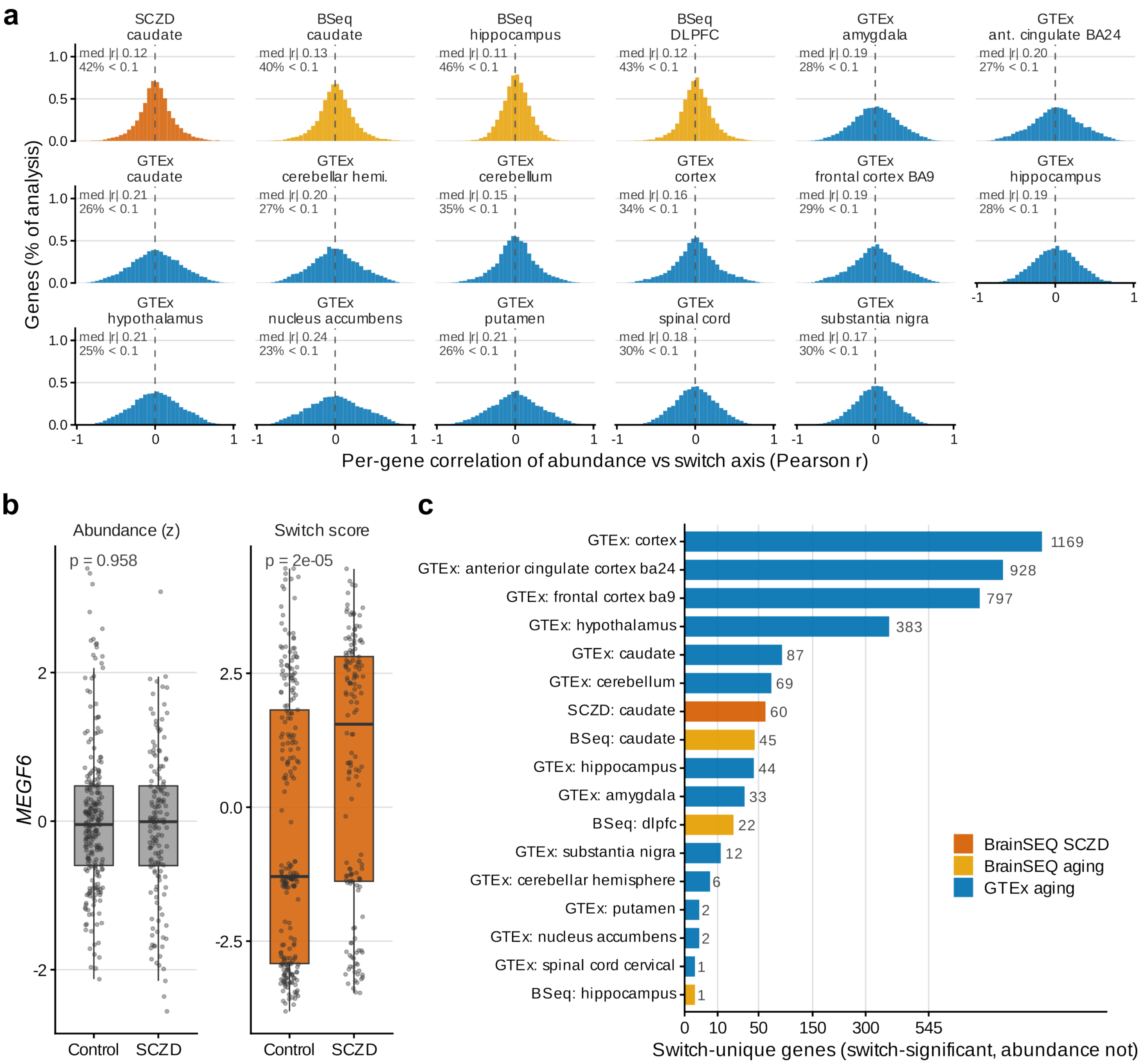

Fig. S12: The isoform-switch and gene-abundance channels are partly non-redundant. a. Distribution of per-gene Pearson correlations between the gene-abundance and isoform-switch coordinates in each of the 17 analyses (12,042–13,222 genes per analysis; the *y* axis is the percentage of that analysis's genes per bin). Each panel is annotated with its median $|r|$ and the percentage of genes with $|r| < 0.1$. **b.** *MEGF6* shows schizophrenia-associated isoform usage in caudate without a detectable conditional gene-abundance association. **c.** Switch-unique genes – those whose switch coordinate is phenotype-associated conditional on abundance while the corresponding abundance test is not – for all 17 analyses, including the five Genotype-Tissue Expression (GTEx) regions that are absent from the composition analyses in Fig. 2c,d because they were not deconvolved. The *x* axis is square-root scaled.

bioRxiv preprint doi: https://doi.org/10.64898/2026.10.04.756439; this version posted October 11, 2026. The copyright holder for this preprint (which was not certified by peer review) is the author/funder, who has granted bioRxiv a license to display the preprint in perpetuity. It is made available under a CC-BY 4.0 International license.

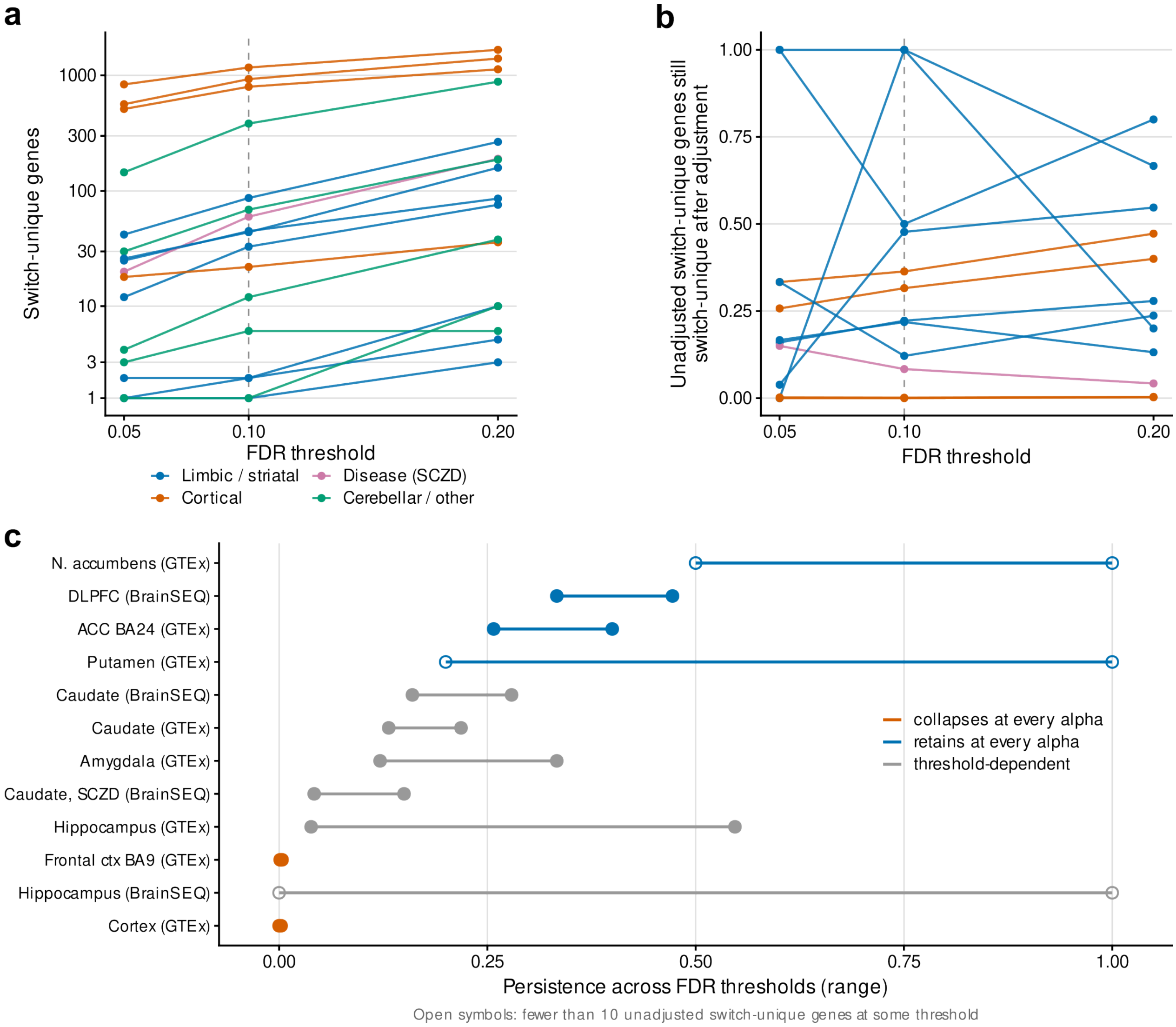

Fig. S13: Sensitivity of the switch-unique classification to the FDR threshold. Genes were reclassified at false discovery rate (FDR) thresholds of 0.05, 0.10 (the value used throughout), and 0.20. Because the Benjamini–Hochberg-adjusted values are computed once across all genes, changing the threshold moves only the cut and no model is refit. **a.** Switch-unique genes per analysis against threshold; the *y* axis is pseudo-log scaled and the dashed line marks 0.10. **b.** Gene-level persistence after cell-composition adjustment against threshold, for the 12 analyses with a composition-adjusted arm. **c.** Range of persistence across the three thresholds per analysis, colored by whether the analysis retains, collapses, or changes call between thresholds. Open symbols mark analyses with fewer than 10 unadjusted switch-unique genes at some threshold, where persistence is a ratio of single-digit counts. The ranking of analyses by switch-unique count is essentially unchanged across thresholds (Spearman ρ = 0.97–0.98 against 0.10), and the two cortical collapses are threshold-independent (persistence ≤ 0.004 at every threshold in Genotype-Tissue Expression (GTEx) cortex and frontal cortex BA9, versus ≥ 0.26 at every threshold in anterior cingulate cortex BA24 and BrainSEQ dorsolateral prefrontal cortex [DLPFC]). The ordering of the intermediate analyses is not threshold-independent, and GTEx hippocampus in particular ranges from 0.04 to 0.55 across the three thresholds.

bioRxiv preprint doi: https://doi.org/10.64898/2026.10.04.756439; this version posted October 11, 2026. The copyright holder for this preprint (which was not certified by peer review) is the author/funder, who has granted bioRxiv a license to display the preprint in perpetuity. It is made available under a CC-BY 4.0 International license.

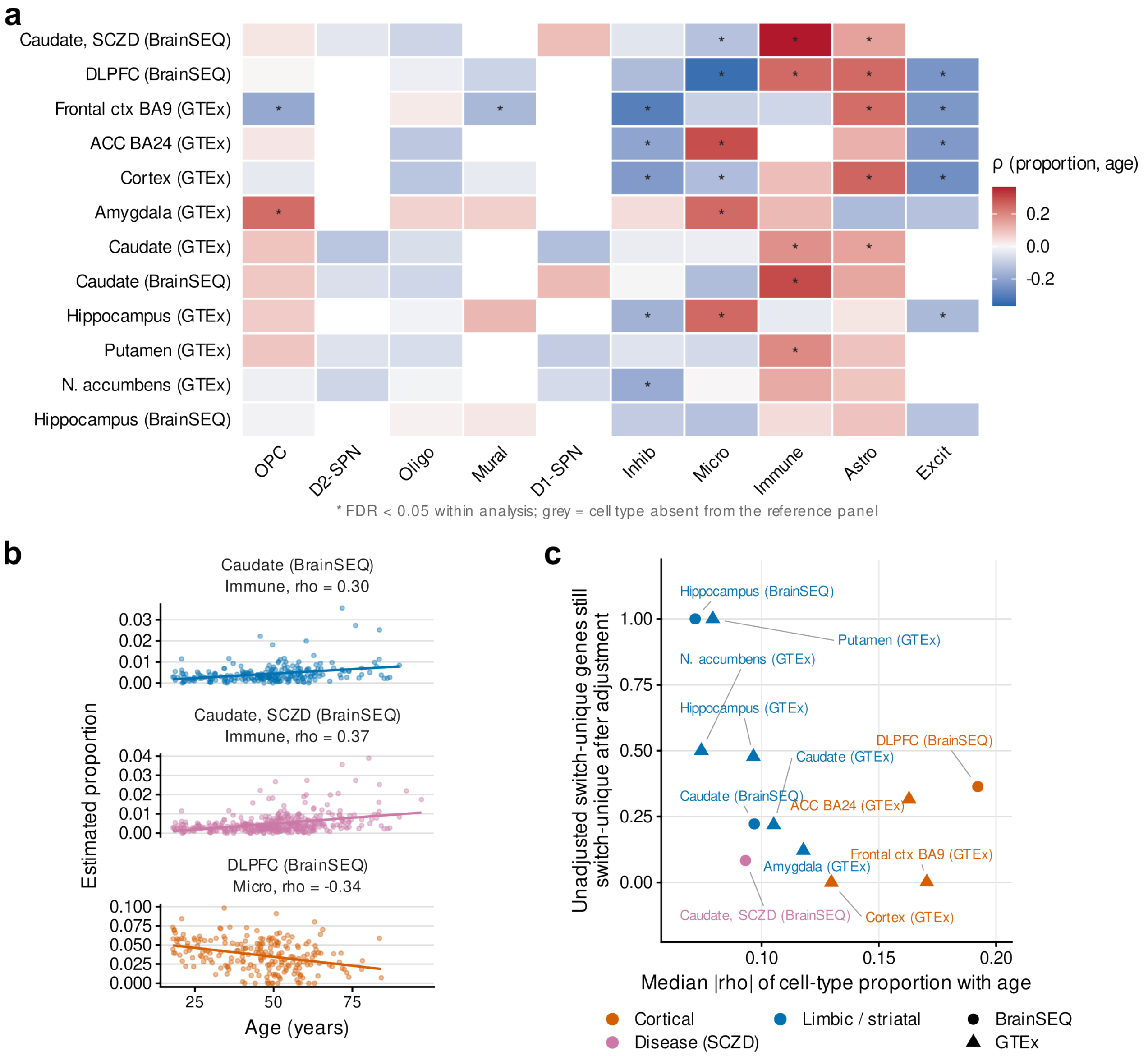

Fig. S14: Estimated cell-type composition is itself age-coupled, but coupling alone does not predict which analyses lose their switch-unique genes. a. Spearman correlation between each estimated cell-type proportion and donor age, per analysis; asterisks mark false discovery rate (FDR) < 0.05 within analysis, and gray cells indicate cell types absent from that region's reference panel. **b.** The sample-level scatter behind the strongest age-coupled cell type in each anatomical class, with an ordinary least-squares line. **c.** Gene-level persistence of the switch-unique set against the analysis's median absolute correlation of cell-type proportion with age. Median absolute correlation is used rather than a count of significant cell types because reference panels differ in size between regions. Cortical analyses carry the most age-coupled composition, yet anterior cingulate cortex BA24 and BrainSEQ dorsolateral prefrontal cortex (DLPFC) retain about a third of their switch-unique genes while frontal cortex BA9 and cortex retain almost none, so composition–age coupling bounds the risk of over-adjustment without resolving it. **d.** Module-by-cell-type marker tests reaching FDR < 0.05 for enrichment and for depletion of cell-type marker genes among module members, pooled by cohort, with the number significant over the number tested. This control does not depend on the covariate model. Only 12 of 2,061 tests showed enrichment and none showed depletion, indicating that modules were not generally enriched for the tested marker sets. This result does not exclude contributions of cellular composition to module structure or establish which biological signals composition adjustment removes.

bioRxiv preprint doi: https://doi.org/10.64898/2026.10.04.756439; this version posted October 11, 2026. The copyright holder for this preprint (which was not certified by peer review) is the author/funder, who has granted bioRxiv a license to display the preprint in perpetuity. It is made available under a CC-BY 4.0 International license.

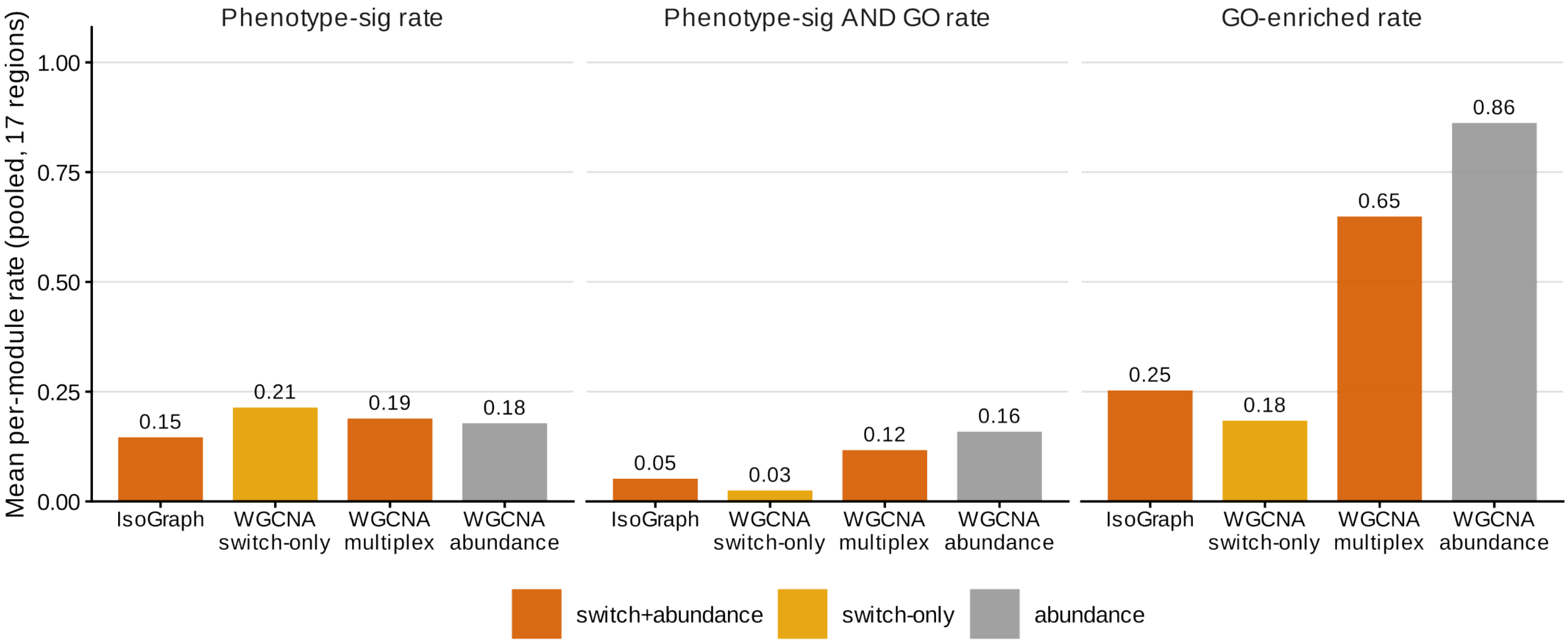

Fig. S15: Per-module rates for the four module sets, on identical samples. Each analysis was partitioned four ways: IsoGraph on switch and abundance features, and classical weighted gene co-expression network analysis (WGCNA) on the same switch-only features, on the same switch and abundance features, and on abundance alone. Bars are the mean per-module rate across the 17 analyses, colored by the feature class each method consumed (switch and abundance, dark orange; switch-only, light orange; abundance, gray). Rates are per-module rather than totals because the four methods partition at different granularity (IsoGraph median 25 modules of median size 108; the WGCNA baselines 8–25 modules of size 159–468), which makes raw counts non-comparable. A module counts as phenotype-significant at module-level false discovery rate (FDR) ≤ 0.1 and as Gene Ontology (GO)-enriched with at least one enriched term. IsoGraph does not carry the highest phenotype-association rate, and the two cohorts rank the methods in opposite orders (IsoGraph 0.176 in BrainSEQ against 0.137 in Genotype-Tissue Expression [GTEx], where 13 of the 17 analyses lie), so the pooled ordering should not be read as a method ranking. This figure is the scope bound on the rest of the results: the defensible contribution of IsoGraph is the content of its modules, not a higher module-level rate.

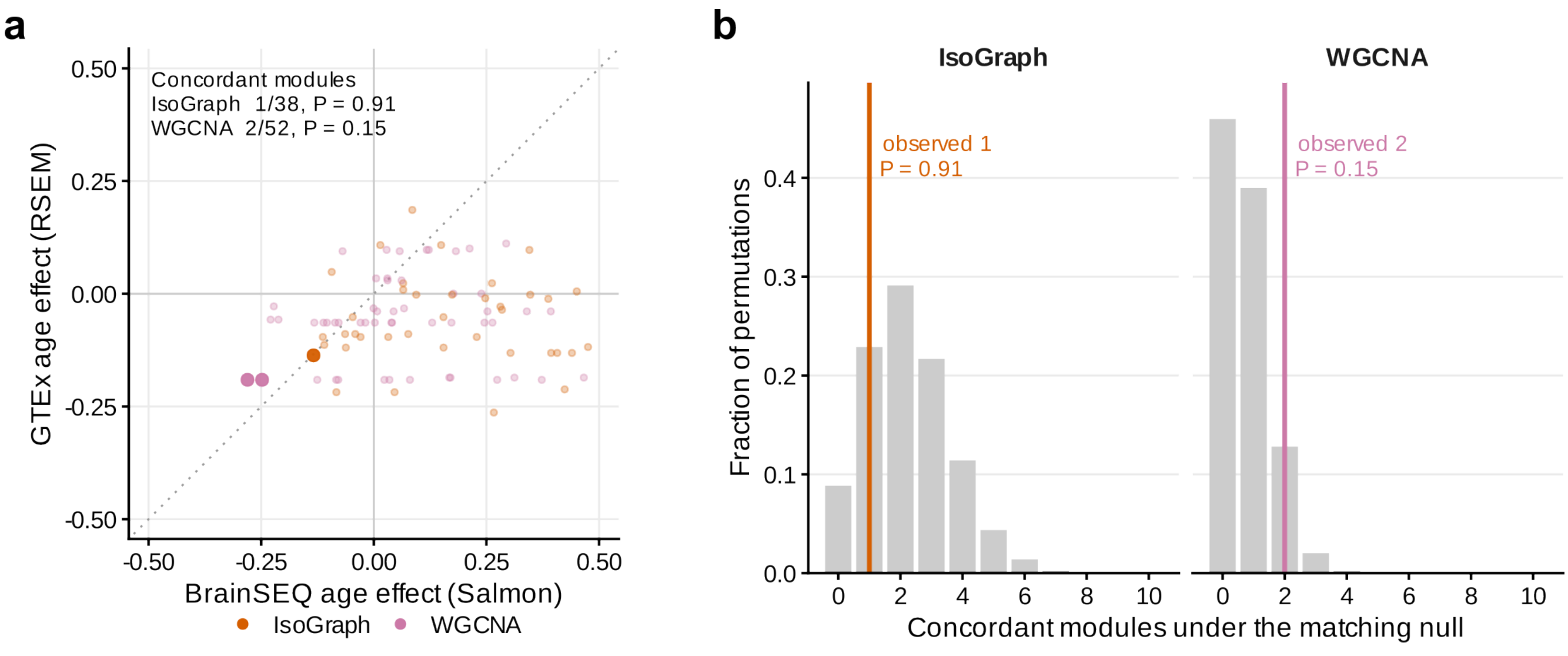

Fig. S16: Cross-cohort module matching by gene membership, and its permutation null. a. Age effect of each matched BrainSEQ module against its best-overlapping Genotype-Tissue Expression (GTEx) module in the anatomically paired region, for both methods (IsoGraph and weighted gene co-expression network analysis [WGCNA]). Larger opaque points are pairs that are nominally significant in both cohorts with a concordant sign. **b.** The observed count of such pairs against the permutation null that holds every age statistic fixed and permutes which target module each source module is matched to. Neither method separates from its null. The two cohorts are quantified by different pipelines – BrainSEQ transcripts by Salmon, GTEx by RSEM – so a module whose age effect fails to carry across them has failed a test that confounds quantification with biology. This comparison is therefore reported as a limitation, and the within-cohort split-half analysis (**Fig. 3d**) carries the reproducibility claim.

bioRxiv preprint doi: https://doi.org/10.64898/2026.10.04.756439; this version posted October 11, 2026. The copyright holder for this preprint (which was not certified by peer review) is the author/funder, who has granted bioRxiv a license to display the preprint in perpetuity. It is made available under a CC-BY 4.0 International license.

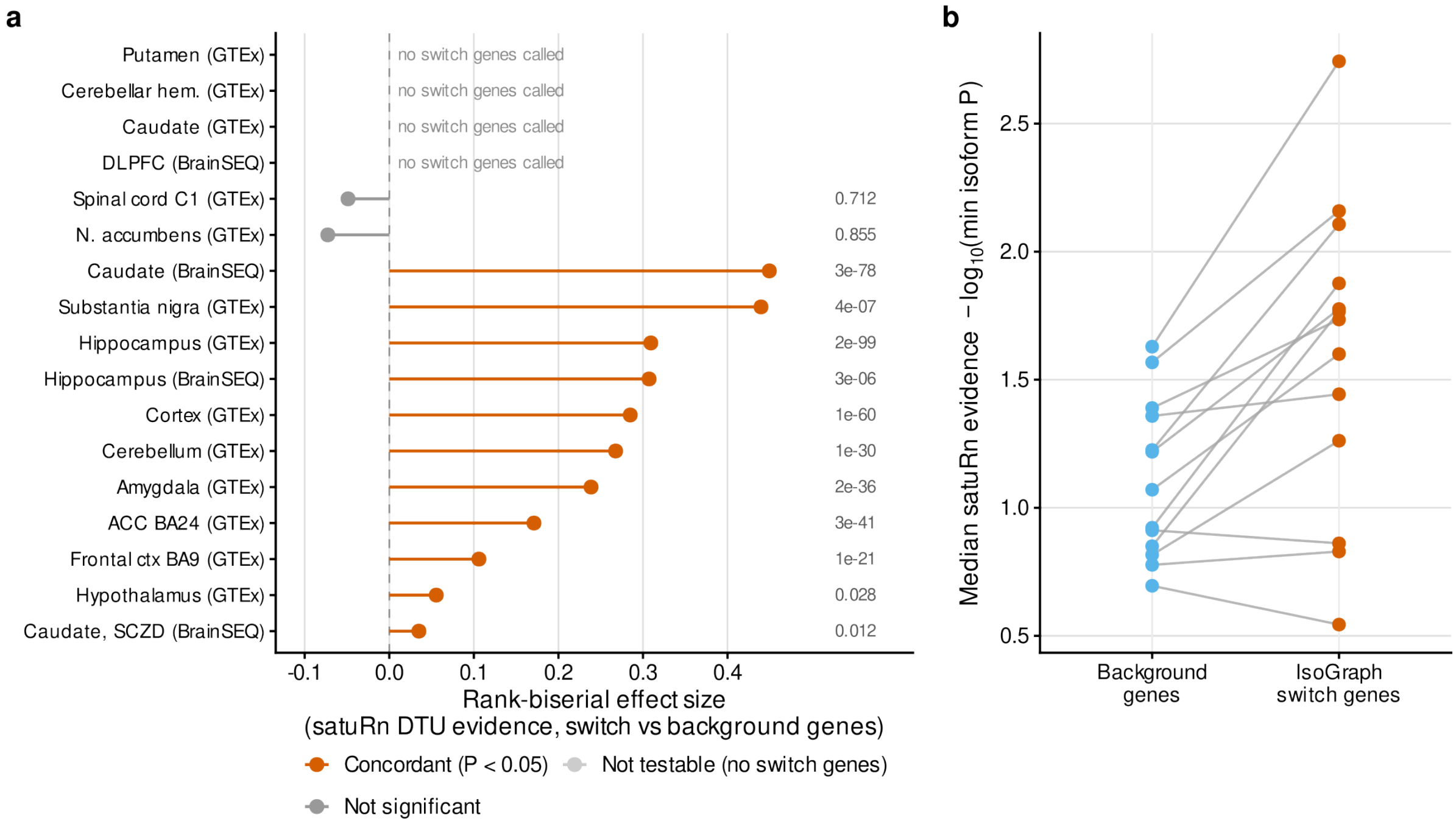

Fig. S17: IsoGraph switch genes carry elevated DTU evidence under satuRn. satuRn, the differential transcript usage (DTU) test run by IsoformSwitchAnalyzeR v2, was fit to the same transcript quantifications with age as a single continuous coefficient, or schizophrenia (SCZD) against control in the BrainSEQ caudate disease analysis, using covariates matched to IsoGraph's discovery design. Each gene's evidence is $-\log_{10}$ of its minimum isoform P. **a.** Rank-biserial effect size comparing that evidence between genes in trait-associated IsoGraph switch modules and all other tested genes, for each of the 17 analyses. Labels give the one-sided Mann–Whitney P. Four analyses had no switch genes; they are shown as not testable rather than as null results, so the denominator is 13. **b.** Median satuRn evidence for background and switch genes in each testable analysis, with lines joining the two values from the same analysis. satuRn's empirical-null false discovery rate (FDR) is not used to binarize the calls: it assumes sparse differential usage, which pervasive age-related DTU in brain violates. Because both methods use the discovery transcript counts, this figure shows agreement between analytical approaches, not independent replication.

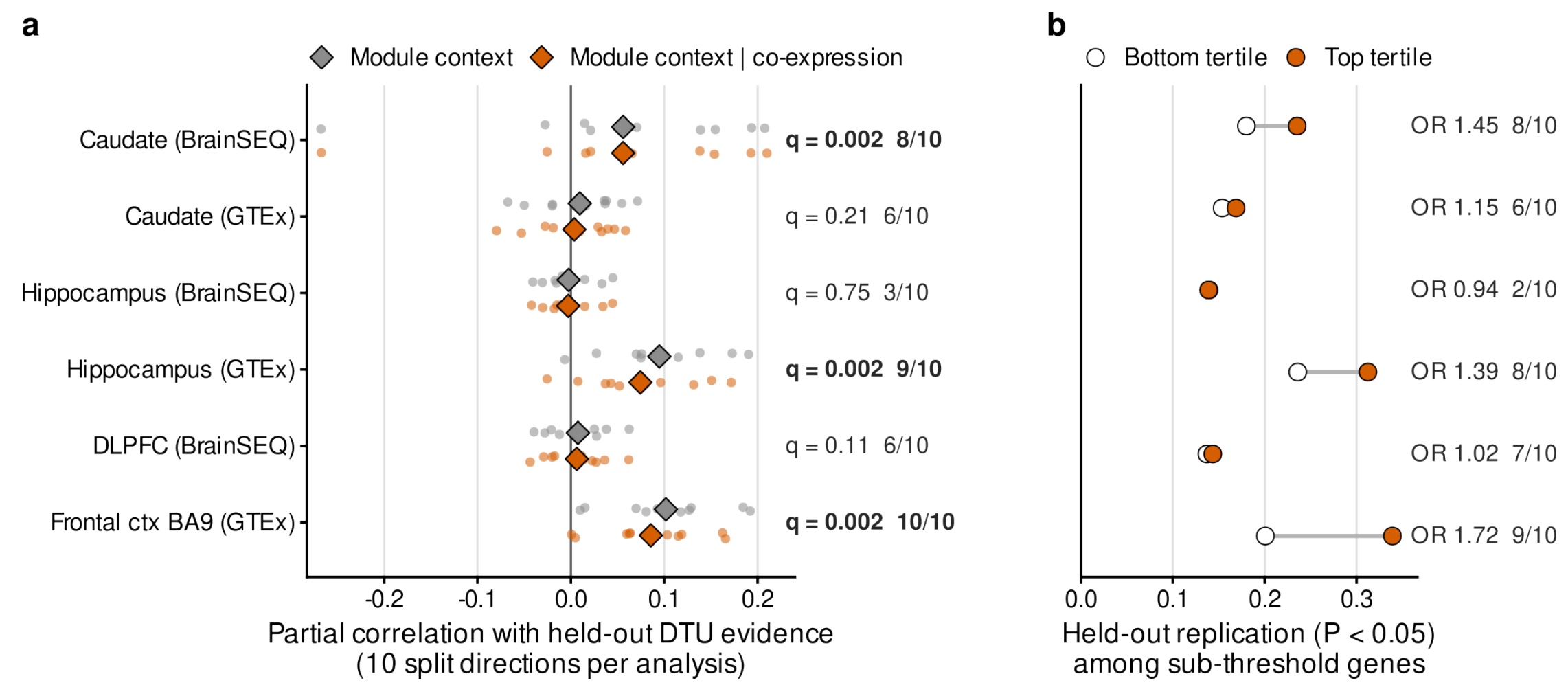

Fig. S18: Module context predicts held-out DTU evidence in three of six analyses. satuRn was fit separately in the two donor halves of five random splits of each of the six anatomically paired cohort-region analyses, giving 10 discovery-to-held-out directions per analysis. **a.** Partial correlation between a gene's module context (the mean discovery-half differential transcript usage [DTU] evidence of its module neighbors, leaving the gene out) and its held-out DTU evidence. The correlation is conditioned on a natural spline of the gene's own discovery evidence, isoform count, expression and minor-isoform usage (gray), and additionally on the discovery evidence of its 50 most co-expressed genes (orange). Small points are individual split directions and diamonds their mean, which is the test statistic. The labels give the Benjamini–Hochberg q of the co-expression-adjusted mean against 1,000 permutations of module labels within technical strata, and the number of positive directions; bold marks $q < 0.05$. **b.** Practical reading of **a**. Among genes that did not meet the discovery DTU criterion (Benjamini–Hochberg $q \geq 0.05$), the fraction reaching $P < 0.05$ in the held-out half, for the bottom (white) and top (orange) tertiles of module context. Labels give the median odds ratio over directions, adjusted for the gene's own discovery evidence and technical covariates, and the number of directions with an odds ratio above 1. This panel is descriptive; **a** is the inference. The held-out threshold is nominal and does not

bioRxiv preprint doi: https://doi.org/10.64898/2026.10.04.756439; this version posted October 11, 2026. The copyright holder for this preprint (which was not certified by peer review) is the author/funder, who has granted bioRxiv a license to display the preprint in perpetuity. It is made available under a CC-BY 4.0 International license.

define false discovery rate (FDR)-significant replication. Module neighbors share discovery-half sampling noise with the gene, and factors shared by both donor halves can contribute to the association.

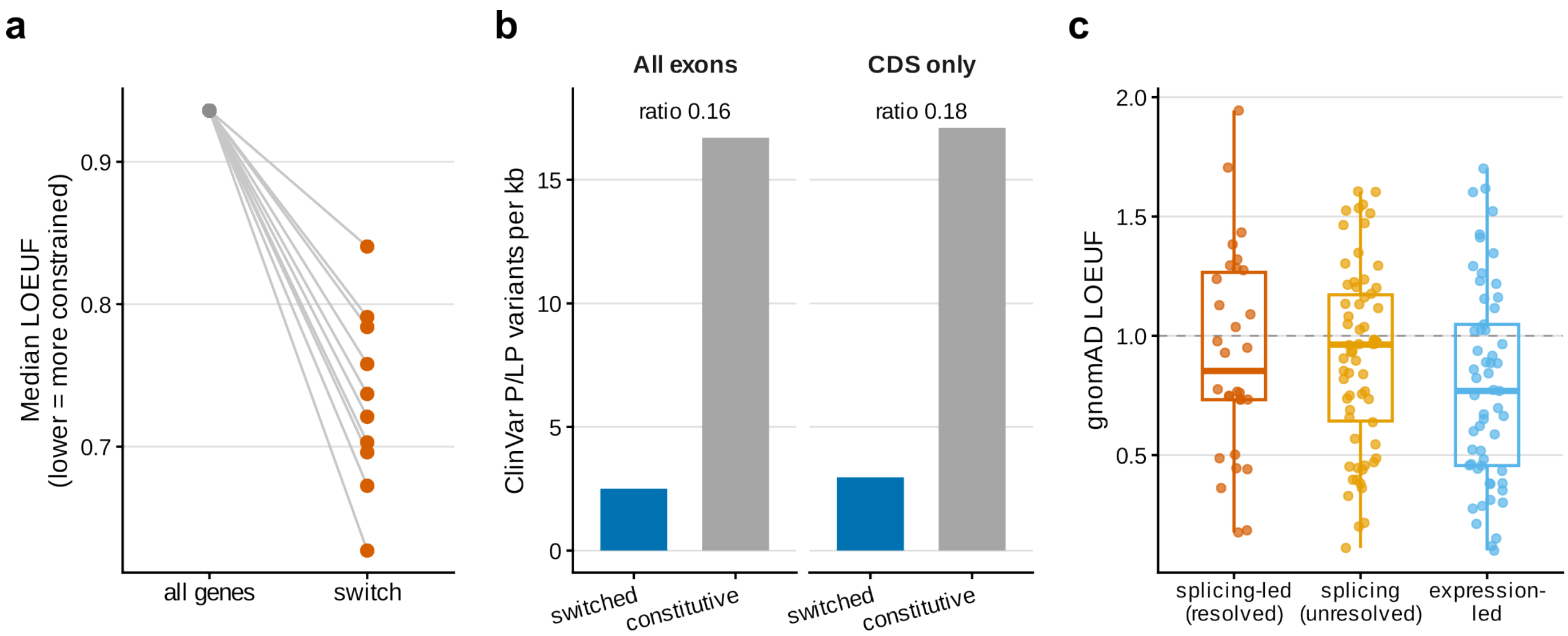

Fig. S19: Switch genes are constrained, but switched exons are depleted of known pathogenic variants. a. Median gnomAD v4.1 loss-of-function observed/expected upper-bound fraction (LOEUF) of switch genes in each of the ten annotatable analyses (orange) against the genome-wide median (gray); lower values indicate stronger loss-of-function constraint. **b.** ClinVar pathogenic or likely-pathogenic entry density per kilobase in exons unique to one member of a switch pair (switched) versus other exons of the same transcripts (constitutive), pooled across analyses, for all exons and for coding exons only; labels give the median within-analysis density ratio. Densities count variant call format (VCF) entries, not unique alleles. **c.** Gene-level LOEUF of colocalization-nominated genes by nomination class (splicing-led with a resolved switch pair, splicing-linked but unresolved, expression-led); this panel is descriptive. The dashed line marks LOEUF 1 (**Table S5**).

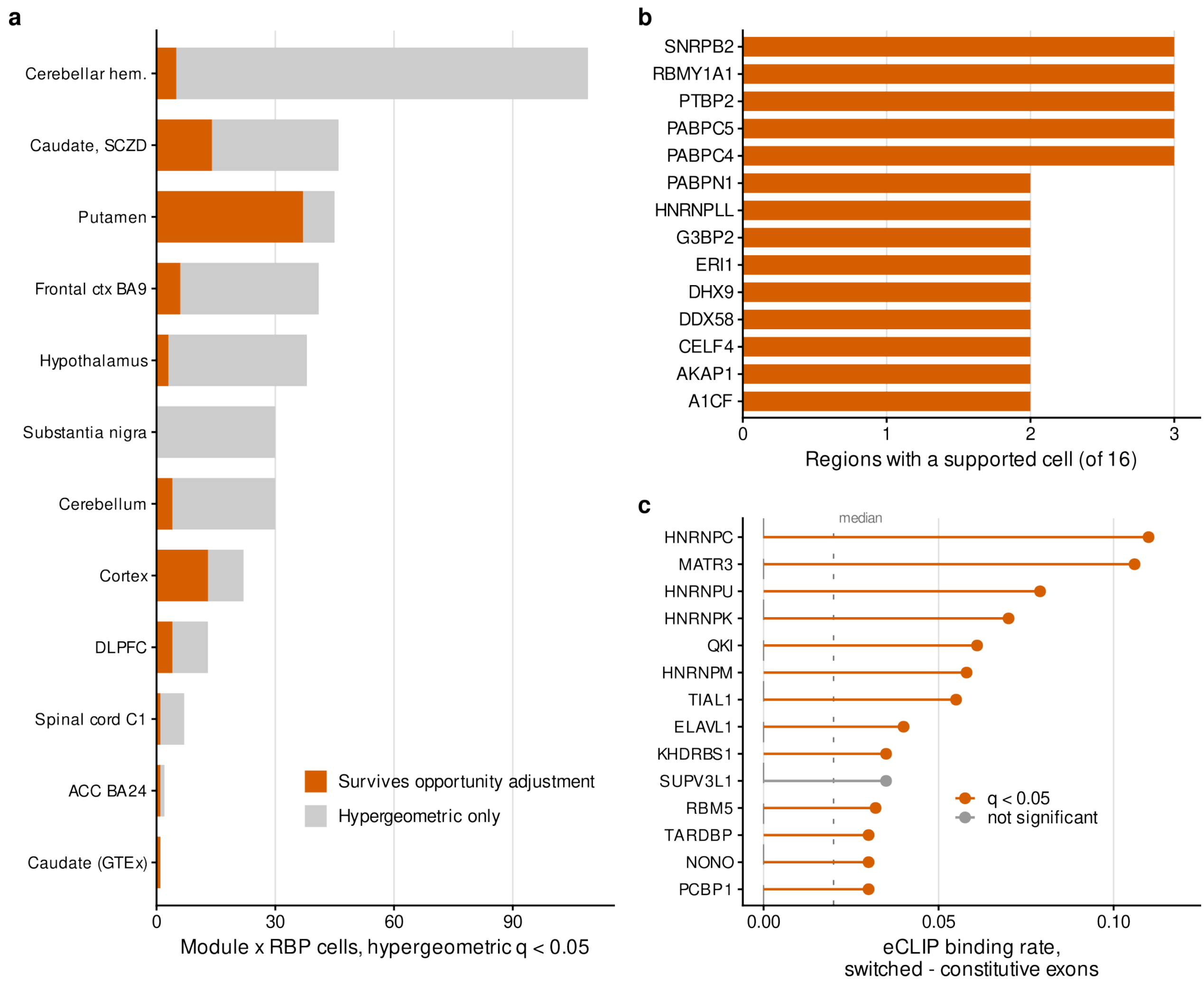

bioRxiv preprint doi: https://doi.org/10.64898/2026.10.04.756439; this version posted October 11, 2026. The copyright holder for this preprint (which was not certified by peer review) is the author/funder, who has granted bioRxiv a license to display the preprint in perpetuity. It is made available under a CC-BY 4.0 International license.

Fig. S20: Candidate RNA-binding-protein regulons of the switch modules. a. Per analysis, module-by-RNA-binding protein (RBP) cells enriched for RBP motif-site switches by the hypergeometric test (Benjamini–Hochberg $q < 0.05$), split by whether they also survive a binomial model adjusting motif opportunity for transcript length, GC content, untranslated-region (UTR) and coding composition, and transcript count (adjusted $q < 0.05$, odds ratio > 1; orange). Analyses with no enriched cell are not shown. **b.** RBPs whose supported cells recur across analyses. **c.** ENCODE enhanced crosslinking and immunoprecipitation (eCLIP; HepG2 and K562 cell lines) binding at switched versus constitutive exon intervals of each RBP's nominated genes, shown as the difference in binding rate for the 14 RBPs with the largest difference. Orange marks false discovery rate (FDR) ≤ 0.05 by an exact McNemar test per RBP, and the dotted line marks the median over all 35 testable RBPs. eCLIP indicates binding capacity in cell lines, not regulation in brain.

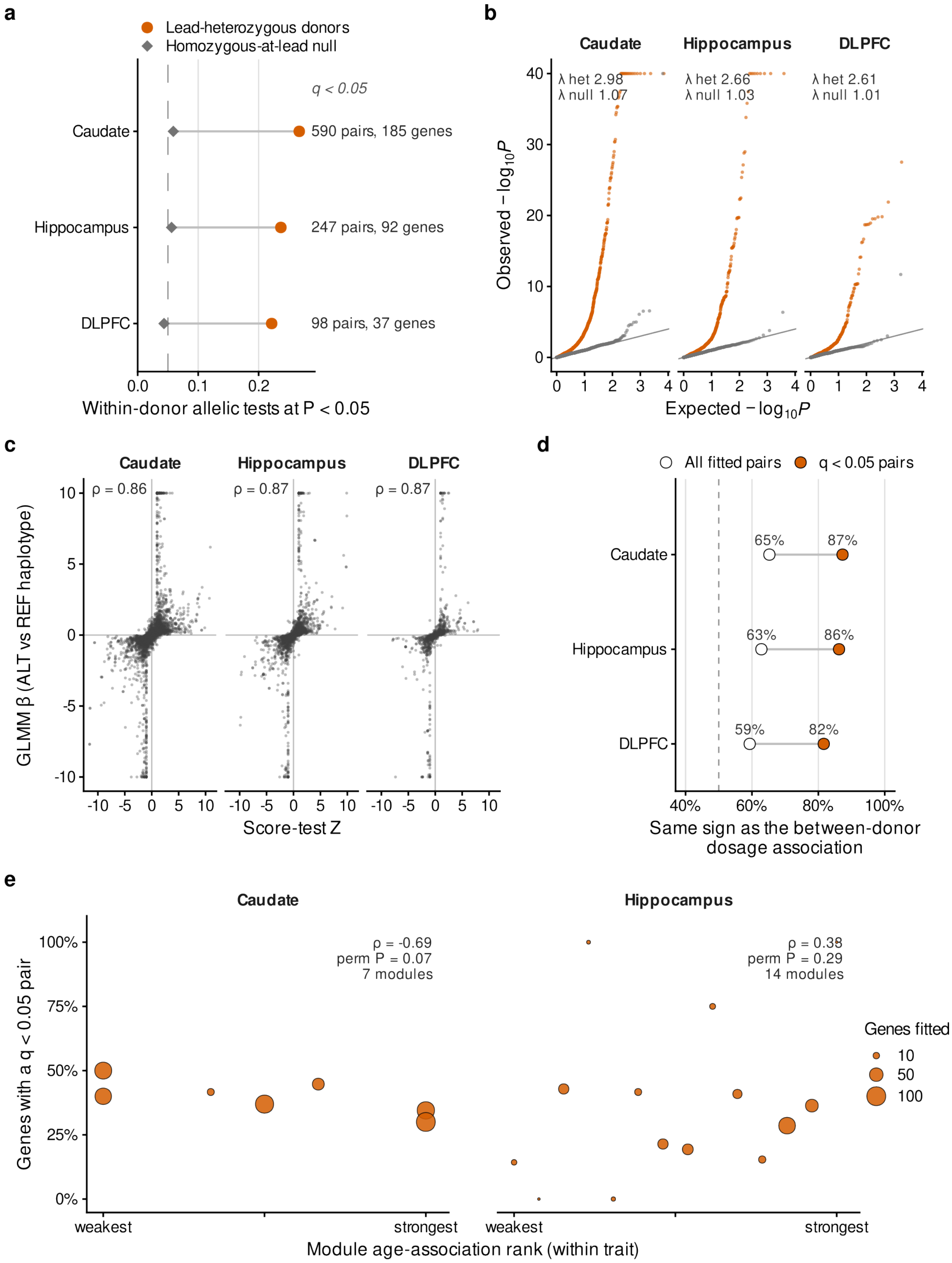

Fig. S21: Calibration and agreement of the within-donor allelic test. BrainSEQ allele-aware junction recount. For each switch pair, fragments crossing isoform-specific junctions were counted on the two haplotypes of donors heterozygous at the switch quantitative trait locus (switch-QTL) lead and compared with a beta-binomial mixed model with a random donor intercept, which absorbs each donor's baseline isoform ratio and cell composition. **a.** As **Fig. 5a**. **b.**

bioRxiv preprint doi: https://doi.org/10.64898/2026.10.04.756439; this version posted October 11, 2026. The copyright holder for this preprint (which was not certified by peer review) is the author/funder, who has granted bioRxiv a license to display the preprint in perpetuity. It is made available under a CC-BY 4.0 International license.

Quantile–quantile plots of all fitted pairs for the lead-heterozygous test (orange) and for the same model fitted to donors homozygous at the lead (gray), with genomic inflation factors. Observed $-\log_{10} P$ is capped at 40 for display. **c.** Mixed-model allelic effect (β, alternate [ALT] against reference [REF] haplotype) against the model-free stratified score-test Z, with Spearman ρ. Fits at $|\beta| = 10$ are quasi-separated (one haplotype carries only one isoform): their sign is informative but their magnitude is not. The 160 pairs without a defined score statistic are not drawn. **d.** Fraction of pairs whose within-donor effect has the same sign as the between-donor association of lead dosage with isoform fraction, estimated from the same reads, over all fitted pairs (white) and pairs at Benjamini–Hochberg $q < 0.05$ (orange); the dashed line marks 50%. **e.** Per IsoGraph module, the fraction of fitted genes with a pair at $q < 0.05$ against the module's age-association rank within its trait; point size gives the genes fitted. ρ is the Spearman correlation, and the permutation P comes from 10,000 permutations of gene-to-module labels that hold module sizes and the number of *cis*-controlled genes (genes with a pair at $q < 0.05$) fixed. Dorsolateral prefrontal cortex (DLPFC) had too few modules per trait to rank. With 7 and 14 modules this panel is descriptive. Pair counts are not independent: pairs of one gene share donors and fragments.

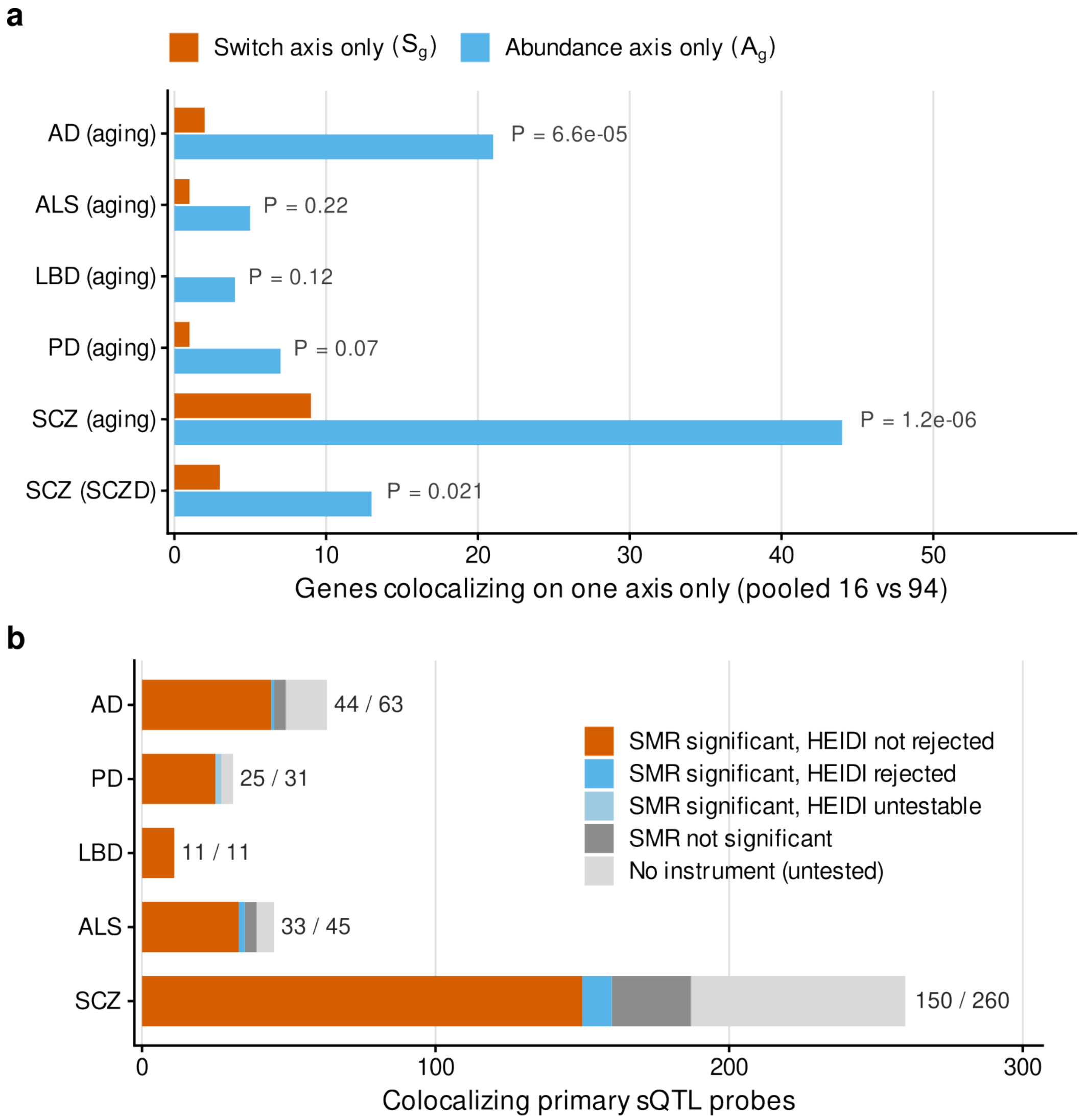

Fig. S22: Corroboration of the colocalization layer. a. In European-ancestry BrainSEQ donors, genes that colocalize with each genome-wide association study (GWAS) on the IsoGraph switch axis only (orange) or the abundance axis only (blue), with exact McNemar P. BrainSEQ is the discovery cohort, so this is same-cohort anchoring rather than replication. **b.** Summary-data-based Mendelian randomization (SMR) and heterogeneity in dependent instruments (HEIDI) tests for the primary confirmatory probe of each colocalizing splicing quantitative trait locus (sQTL) gene, by trait. Bars count probes that SMR supports with HEIDI not rejecting a single shared variant (orange), probes that HEIDI rejects or cannot test (blue), probes that SMR leaves non-significant (dark gray) and probes without an instrument at QTL $P < 5 \times 10^{-8}$ (light gray), which are untested rather than negative. Labels give supported over all probes. HEIDI is underpowered at Genotype-Tissue Expression (GTEx) brain sample sizes, so non-rejection is not evidence of a shared variant.

bioRxiv preprint doi: https://doi.org/10.64898/2026.10.04.756439; this version posted October 11, 2026. The copyright holder for this preprint (which was not certified by peer review) is the author/funder, who has granted bioRxiv a license to display the preprint in perpetuity. It is made available under a CC-BY 4.0 International license.

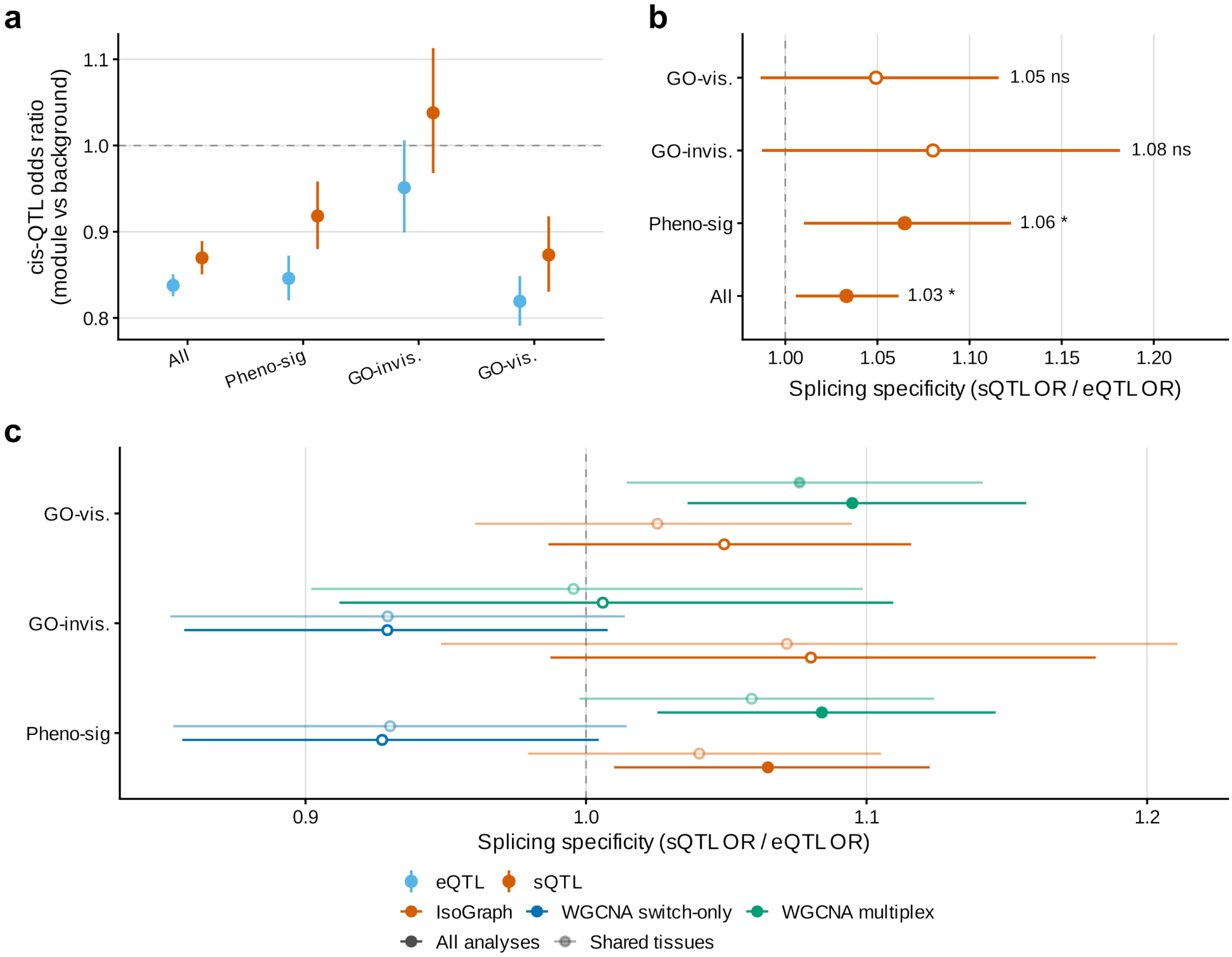

Fig. S23: Splicing-QTL specificity of module genes. a. Pooled odds ratios (fixed-effect inverse-variance meta-analysis across analyses) for *cis*-expression quantitative trait locus (eQTL; blue) and *cis*-splicing QTL (sQTL; orange) status of IsoGraph module genes relative to the tested background, by module set: all modules (17 analyses), phenotype-associated modules (10), Gene Ontology (GO)-invisible modules (7), and GO-visible modules (9). Both QTL classes are depleted. **b.** The paired within-analysis ratio of the sQTL to the eQTL odds ratio, the primary estimand; bars are 95% confidence intervals and filled points have $P < 0.05$. **c.** The same ratio for IsoGraph (orange), weighted gene co-expression network analysis (WGCNA) on the same switch-only features (blue), and WGCNA on the same switch-plus-abundance features (green), over all analyses (dark) and over the analyses shared by all three methods (light). The contrast is present for both methods that consume the paired features and absent for the switch-only representation. Logistic models adjusted for *cis*-variant count, gene length, retained isoform count and intron-group size (**Tables S6** and **S7**).

bioRxiv preprint doi: https://doi.org/10.64898/2026.10.04.756439; this version posted October 11, 2026. The copyright holder for this preprint (which was not certified by peer review) is the author/funder, who has granted bioRxiv a license to display the preprint in perpetuity. It is made available under a CC-BY 4.0 International license.

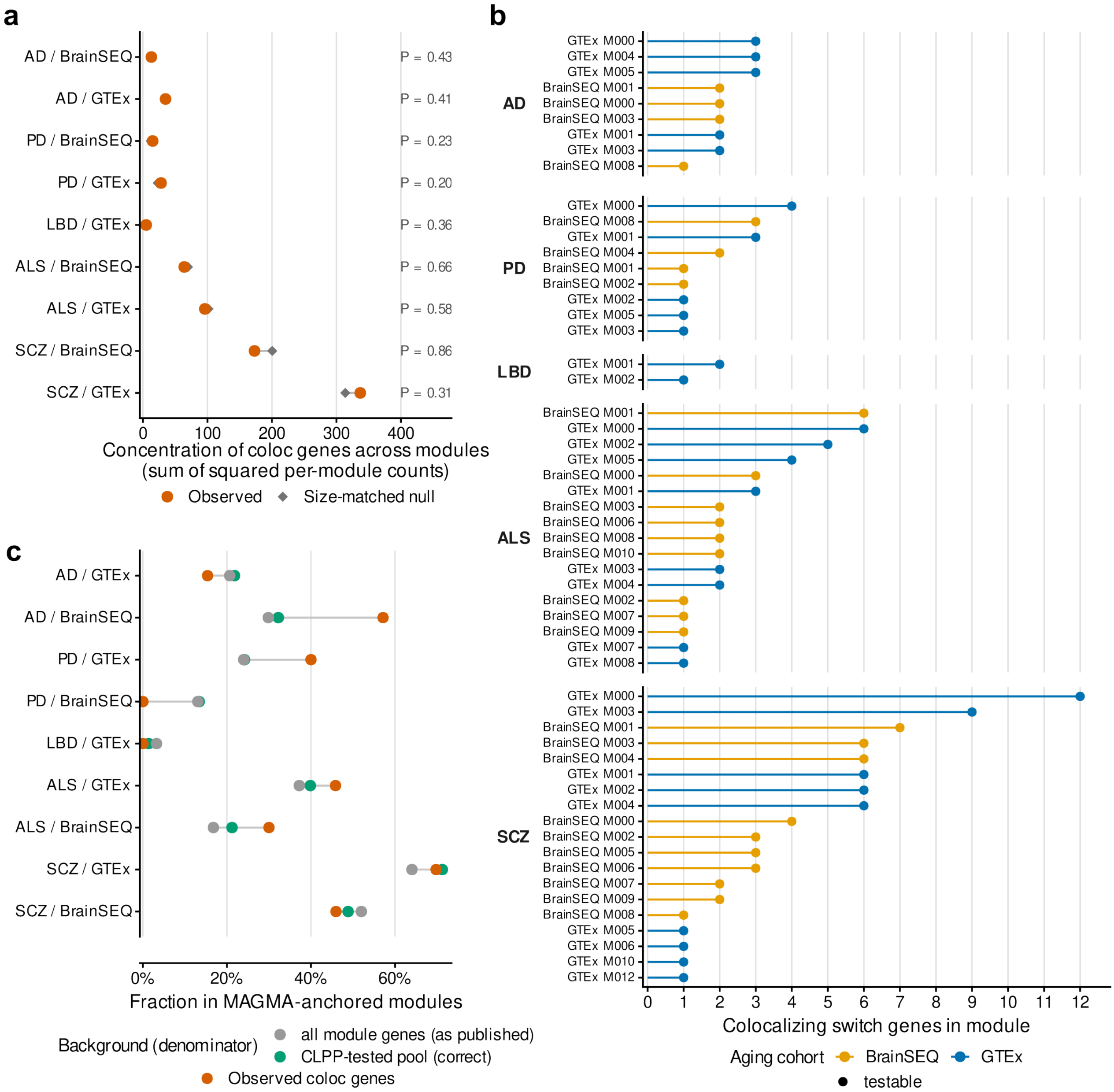

Fig. S24: Posterior-overlap-supported genes show no detectable concentration in IsoGraph modules. a. For each trait and aging cohort, the concentration of posterior-overlap-supported module-member genes across modules (sum of squared per-module counts; orange) against a null that redraws the same number of genes from the overlap-score-tested pool with module sizes fixed (gray, null mean). *P* values are empirical. Dementia with Lewy bodies (LBD) had no overlap-supported genes in BrainSEQ and is not tested. **b.** Overlap-supported genes per module, by trait and cohort (BrainSEQ, yellow; Genotype-Tissue Expression [GTEx], blue). **c.** Fraction of overlap-supported genes that fall in MAGMA-anchored modules (modules with a competitive gene-set $P < 0.05$; orange), against two backgrounds: all module genes (gray) and the overlap-score-tested pool (green). Genes enter the overlap-score screen partly through the same genome-wide association study (GWAS) that defines MAGMA anchoring, so the tested pool is already enriched for anchored modules and is the correct denominator. Against it, no trait shows enrichment.

bioRxiv preprint doi: https://doi.org/10.64898/2026.10.04.756439; this version posted October 11, 2026. The copyright holder for this preprint (which was not certified by peer review) is the author/funder, who has granted bioRxiv a license to display the preprint in perpetuity. It is made available under a CC-BY 4.0 International license.

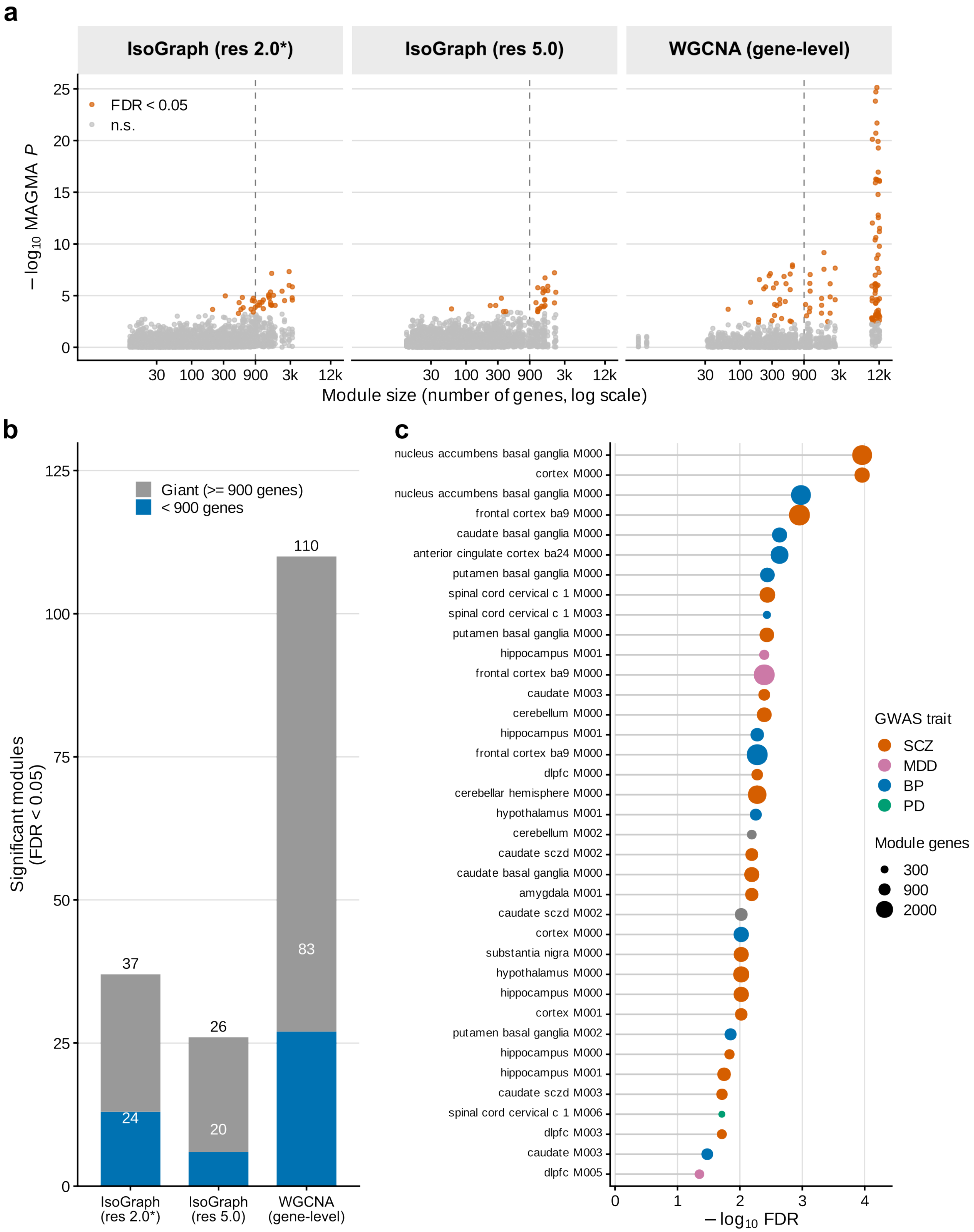

Fig. S25: Significant module-level GWAS associations are concentrated among large modules. a. MAGMA competitive gene-set $-\log_{10} P$ against module size for IsoGraph modules at the production Leiden resolution 2.0, the resolution-5.0 sensitivity arm, and classical abundance-only weighted gene co-expression network analysis (WGCNA), over eight genome-wide association study (GWAS) traits; orange marks false discovery rate (FDR) < 0.05 and the dashed line the 900-gene cutoff. **b.** FDR-significant module-by-trait sets split by size: 24 of 37 IsoGraph sets at resolution 2.0, 20 of 26 at resolution 5.0, and 83 of 110 WGCNA sets fall in modules of at least 900 genes. **c.** The 37 FDR-significant IsoGraph sets at resolution 2.0 by $-\log_{10}$ FDR, colored by trait (gray, amyotrophic lateral sclerosis [ALS]) and sized by module genes. Module identifiers are specific to this partition (**Data** S24).

bioRxiv preprint doi: https://doi.org/10.64898/2026.10.04.756439; this version posted October 11, 2026. The copyright holder for this preprint (which was not certified by peer review) is the author/funder, who has granted bioRxiv a license to display the preprint in perpetuity. It is made available under a CC-BY 4.0 International license.

Supplementary Tables

Table S1: Synthetic benchmark performance across scenarios and methods. Performance metrics are reported as means with 95% confidence intervals (CIs) in brackets. Runtime is reported as the median in seconds. *N* is the number of benchmark runs summarized in each row. Module recovery is the best-match Jaccard similarity between planted and inferred modules (1 = perfect recovery); switch detection is the recall of planted switching genes; the non-switching gene module rate is the fraction of non-switching genes assigned to modules, and lower values indicate better performance. Methods are the IsoGraph backends (Baseline, Latent, Graph, and VAE), a Spearman-correlation network with Leiden clustering (Spearman-Leiden), and WGCNA. ARI, adjusted Rand index; CPU, central processing unit; NA, unavailable; VAE, variational autoencoder; WGCNA, weighted gene co-expression network analysis.

Scenario	Method	Compute	*N*	Module recovery, mean (95% CI)	ARI (planted), mean (95% CI)	ARI (assigned genes only), mean (95% CI)	Homogeneity, mean (95% CI)	Completeness, mean (95% CI)	Switch detection, mean (95% CI)	Non-switching gene module rate (lower is better), mean (95% CI)	Runtime (s), median
Idealized switching	IsoGraph Baseline	CPU	360	0.074 [0.070, 0.077]	0.001 [0.001, 0.002]	0.001 [0.001, 0.001]	0.118 [0.100, 0.137]	0.684 [0.650, 0.719]	0.898 [0.881, 0.914]	0.297 [0.282, 0.311]	0.7
Idealized switching	IsoGraph Latent	CPU	360	0.217 [0.183, 0.253]	0.468 [0.408, 0.530]	0.996 [0.991, 1.000]	0.999 [0.998, 1.000]	0.683 [0.650, 0.717]	0.217 [0.182, 0.253]	0.000 [0.000, 0.000]	5.7
Idealized switching	IsoGraph Graph	CPU	360	0.300 [0.265, 0.336]	0.590 [0.555, 0.626]	0.995 [0.989, 1.000]	0.999 [0.998, 1.000]	0.706 [0.683, 0.730]	0.300 [0.265, 0.336]	0.000 [0.000, 0.000]	6.5
Idealized switching	IsoGraph VAE	CPU	360	0.968 [0.961, 0.974]	0.998 [0.996, 1.000]	0.998 [0.996, 1.000]	0.999 [0.998, 1.000]	1.000 [1.000, 1.000]	1.000 [1.000, 1.000]	0.021 [0.018, 0.024]	4.1
Idealized switching	Spearman-Leiden	CPU	360	0.632 [0.628, 0.637]	0.615 [0.611, 0.620]	0.630 [0.624, 0.636]	0.989 [0.987, 0.991]	0.736 [0.733, 0.739]	0.985 [0.982, 0.988]	0.089 [0.086, 0.092]	1
Idealized switching	WGCNA	CPU	360	0.572 [0.557, 0.586]	0.649 [0.645, 0.653]	0.669 [0.663, 0.674]	0.991 [0.988, 0.994]	0.766 [0.764, 0.768]	0.980 [0.976, 0.984]	0.232 [0.192, 0.274]	3.4
Noise stress	IsoGraph Baseline	CPU	320	0.057 [0.056, 0.057]	0.000 [-0.000, 0.000]	0.000 [0.000, 0.000]	0.000 [0.000, 0.000]	0.997 [0.991, 1.000]	1.000 [1.000, 1.000]	0.419 [0.410, 0.428]	0.7
Noise stress	IsoGraph Latent	CPU	320	0.113 [0.101, 0.126]	0.078 [0.068, 0.088]	1.000 [1.000, 1.000]	1.000 [1.000, 1.000]	0.473 [0.471, 0.476]	0.111 [0.099, 0.123]	0.000 [0.000, 0.000]	6.3
Noise stress	IsoGraph Graph	CPU	320	0.500 [0.490, 0.511]	0.421 [0.411, 0.430]	1.000 [1.000, 1.000]	1.000 [1.000, 1.000]	0.580 [0.576, 0.584]	0.497 [0.487, 0.508]	0.000 [0.000, 0.000]	6.3
Noise stress	IsoGraph VAE	CPU	320	0.974 [0.971, 0.977]	1.000 [0.999, 1.000]	1.000 [0.999, 1.000]	1.000 [0.999, 1.000]	1.000 [1.000, 1.000]	1.000 [1.000, 1.000]	0.020 [0.018, 0.022]	4.1
Noise stress	Spearman-Leiden	CPU	320	0.636 [0.631, 0.641]	0.614 [0.609, 0.618]	0.615 [0.611, 0.620]	0.991 [0.989, 0.993]	0.732 [0.730, 0.734]	0.998 [0.998, 0.999]	0.092 [0.089, 0.094]	1
Noise stress	WGCNA	CPU	320	0.629 [0.619, 0.638]	0.663 [0.659, 0.666]	0.665 [0.662, 0.669]	1.000 [1.000, 1.000]	0.768 [0.767, 0.769]	0.997 [0.997, 0.998]	0.046 [0.027, 0.067]	3.4
Feature interactions	IsoGraph Baseline	CPU	300	0.052 [0.051, 0.053]	0.000 [0.000, 0.000]	0.000 [0.000, 0.000]	0.000 [0.000, 0.000]	1.000 [1.000, 1.000]	1.000 [1.000, 1.000]	0.489 [0.476, 0.503]	0.7
Feature interactions	IsoGraph Latent	CPU	300	0.326 [0.312, 0.340]	0.238 [0.225, 0.250]	0.766 [0.750, 0.782]	1.000 [1.000, 1.000]	0.532 [0.528, 0.537]	0.437 [0.416, 0.457]	0.000 [0.000, 0.000]	6.2

bioRxiv preprint doi: https://doi.org/10.64898/2026.10.04.756439; this version posted October 11, 2026. The copyright holder for this preprint (which was not certified by peer review) is the author/funder, who has granted bioRxiv a license to display the preprint in perpetuity. It is made available under a CC-BY 4.0 International license.

Scenario	Method	Compute	*N*	Module recovery, mean (95% CI)	ARI (planted), mean (95% CI)	ARI (assigned genes only), mean (95% CI)	Homogeneity, mean (95% CI)	Completeness, mean (95% CI)	Switch detection, mean (95% CI)	Non-switching gene module rate (lower is better), mean (95% CI)	Runtime (s), median
Feature interactions	IsoGraph Graph	CPU	300	0.482 [0.473, 0.491]	0.385 [0.375, 0.396]	0.856 [0.844, 0.868]	1.000 [1.000, 1.000]	0.580 [0.576, 0.584]	0.565 [0.553, 0.576]	0.000 [0.000, 0.000]	6.8
Feature interactions	IsoGraph VAE	CPU	300	0.769 [0.739, 0.799]	0.670 [0.626, 0.715]	0.671 [0.626, 0.715]	0.786 [0.755, 0.817]	0.847 [0.825, 0.869]	0.999 [0.999, 0.999]	0.009 [0.008, 0.010]	5.3
Feature interactions	Spearman-Leiden	CPU	300	0.449 [0.428, 0.469]	0.335 [0.307, 0.363]	0.336 [0.308, 0.364]	0.628 [0.590, 0.665]	0.560 [0.539, 0.581]	0.998 [0.998, 0.999]	0.089 [0.087, 0.092]	0.9
Feature interactions	WGCNA	CPU	300	0.476 [0.456, 0.496]	0.425 [0.397, 0.453]	0.427 [0.400, 0.455]	0.795 [0.761, 0.829]	0.614 [0.593, 0.636]	0.990 [0.988, 0.992]	0.088 [0.058, 0.118]	3.1
Non-switching background	IsoGraph Baseline	CPU	80	0.054 [0.047, 0.062]	0.002 [0.000, 0.004]	0.002 [0.000, 0.004]	0.018 [0.011, 0.027]	0.835 [0.769, 0.893]	0.990 [0.986, 0.994]	0.342 [0.325, 0.359]	0.7
Non-switching background	IsoGraph Latent	CPU	80	0.312 [0.237, 0.393]	0.481 [0.388, 0.571]	0.890 [0.829, 0.945]	0.972 [0.954, 0.987]	0.727 [0.677, 0.776]	0.346 [0.269, 0.426]	0.000 [0.000, 0.000]	6.2
Non-switching background	IsoGraph Graph	CPU	80	0.382 [0.314, 0.453]	0.555 [0.493, 0.621]	0.865 [0.787, 0.935]	0.965 [0.941, 0.985]	0.736 [0.703, 0.771]	0.420 [0.354, 0.487]	0.000 [0.000, 0.000]	6.9
Non-switching background	IsoGraph VAE	CPU	80	0.841 [0.778, 0.898]	0.911 [0.849, 0.964]	0.911 [0.849, 0.964]	0.924 [0.867, 0.972]	1.000 [1.000, 1.000]	1.000 [1.000, 1.000]	0.109 [0.067, 0.157]	4.6
Non-switching background	Spearman-Leiden	CPU	80	0.629 [0.609, 0.649]	0.609 [0.592, 0.628]	0.698 [0.667, 0.731]	0.990 [0.985, 0.994]	0.761 [0.750, 0.772]	0.905 [0.870, 0.937]	0.093 [0.088, 0.099]	0.9
Non-switching background	WGCNA	CPU	80	0.535 [0.499, 0.570]	0.633 [0.611, 0.655]	0.650 [0.626, 0.673]	0.985 [0.976, 0.993]	0.784 [0.776, 0.793]	0.983 [0.976, 0.990]	0.342 [0.242, 0.442]	4.2
Unequal abundance	IsoGraph Baseline	CPU	80	0.082 [0.071, 0.095]	0.013 [0.006, 0.022]	0.220 [0.141, 0.306]	0.274 [0.184, 0.372]	0.748 [0.687, 0.809]	0.766 [0.675, 0.848]	0.240 [0.201, 0.278]	0.6
Unequal abundance	IsoGraph Latent	CPU	80	0.152 [0.131, 0.173]	0.086 [0.071, 0.102]	1.000 [1.000, 1.000]	1.000 [1.000, 1.000]	0.476 [0.472, 0.480]	0.148 [0.128, 0.170]	0.000 [0.000, 0.000]	5.8
Unequal abundance	IsoGraph Graph	CPU	80	0.428 [0.395, 0.458]	0.336 [0.307, 0.364]	1.000 [1.000, 1.000]	1.000 [1.000, 1.000]	0.554 [0.544, 0.564]	0.425 [0.393, 0.456]	0.000 [0.000, 0.000]	5.7
Unequal abundance	IsoGraph VAE	CPU	80	0.780 [0.707, 0.849]	0.818 [0.738, 0.894]	0.818 [0.738, 0.894]	0.833 [0.755, 0.904]	0.999 [0.998, 1.000]	1.000 [1.000, 1.000]	0.151 [0.107, 0.198]	4
Unequal abundance	Spearman-Leiden	CPU	80	0.620 [0.611, 0.628]	0.604 [0.596, 0.611]	0.606 [0.598, 0.614]	0.990 [0.986, 0.993]	0.730 [0.726, 0.734]	0.998 [0.997, 0.999]	0.096 [0.091, 0.101]	0.9
Unequal abundance	WGCNA	CPU	80	0.553 [0.524, 0.580]	0.600 [0.579, 0.620]	0.605 [0.584, 0.624]	0.999 [0.998, 1.000]	0.737 [0.726, 0.747]	0.994 [0.991, 0.997]	0.093 [0.050, 0.141]	4

bioRxiv preprint doi: https://doi.org/10.64898/2026.10.04.756439; this version posted October 11, 2026. The copyright holder for this preprint (which was not certified by peer review) is the author/funder, who has granted bioRxiv a license to display the preprint in perpetuity. It is made available under a CC-BY 4.0 International license.

Scenario	Method	Compute	*N*	Module recovery, mean (95% CI)	ARI (planted), mean (95% CI)	ARI (assigned genes only), mean (95% CI)	Homogeneity, mean (95% CI)	Completeness, mean (95% CI)	Switch detection, mean (95% CI)	Non-switching gene module rate (lower is better), mean (95% CI)	Runtime (s), median
Negative control	IsoGraph Baseline	CPU	40	0.000 [0.000, 0.000]	NA [NA, NA]	NA [NA, NA]	NA [NA, NA]	NA [NA, NA]	0.000 [0.000, 0.000]	0.000 [0.000, 0.000]	1.4
Negative control	IsoGraph Latent	CPU	40	0.000 [0.000, 0.000]	NA [NA, NA]	NA [NA, NA]	NA [NA, NA]	NA [NA, NA]	0.000 [0.000, 0.000]	0.000 [0.000, 0.000]	25.8
Negative control	IsoGraph Graph	CPU	40	0.000 [0.000, 0.000]	NA [NA, NA]	NA [NA, NA]	NA [NA, NA]	NA [NA, NA]	0.000 [0.000, 0.000]	0.000 [0.000, 0.000]	21
Negative control	IsoGraph VAE	CPU	40	0.054 [0.017, 0.100]	NA [NA, NA]	NA [NA, NA]	NA [NA, NA]	NA [NA, NA]	0.925 [0.825, 1.000]	0.790 [0.687, 0.884]	4.7
Negative control	Spearman-Leiden	CPU	40	0.013 [0.000, 0.037]	NA [NA, NA]	NA [NA, NA]	NA [NA, NA]	NA [NA, NA]	0.025 [0.000, 0.075]	0.095 [0.089, 0.101]	1
Negative control	WGCNA	CPU	40	0.405 [0.359, 0.446]	NA [NA, NA]	NA [NA, NA]	NA [NA, NA]	NA [NA, NA]	1.000 [1.000, 1.000]	1.000 [1.000, 1.000]	24.3

Table S2: Runtime and memory use across network sizes and compute settings. Runtime, peak host RAM, and peak reserved GPU VRAM are reported as medians with interquartile ranges (IQRs) in brackets. *N* is the number of benchmark runs summarized in each row. Runtime is measured in seconds and memory in gigabytes (GB). NA indicates that GPU VRAM measurements are not applicable to CPU runs. CPU, central processing unit; GPU, graphics processing unit; RAM, random-access memory; VAE, variational autoencoder; VRAM, video RAM; WGCNA, weighted gene co-expression network analysis.

Genes (*n*)	Method	Compute	*N*	Runtime (s), median [IQR]	Peak host RAM (GB), median [IQR]	Peak reserved GPU VRAM (GB), median [IQR]
1000	IsoGraph VAE	CPU	30	6.3 [5.8, 7.4]	0.9 [0.8, 1.0]	NA
1000	IsoGraph VAE GPU	GPU	30	4.1 [3.8, 4.5]	1.2 [1.2, 1.2]	0.0 [0.0, 0.1]
1000	WGCNA	CPU	30	6.7 [4.7, 8.1]	0.7 [0.6, 0.7]	NA
3000	IsoGraph VAE	CPU	30	28.2 [18.0, 35.0]	1.1 [1.1, 1.2]	NA
3000	IsoGraph VAE GPU	GPU	30	7.9 [7.3, 8.6]	1.4 [1.3, 1.5]	0.2 [0.1, 0.3]
3000	WGCNA	CPU	30	17.8 [11.3, 26.4]	1.0 [1.0, 1.8]	NA
6000	IsoGraph VAE	CPU	30	36.7 [29.3, 84.0]	1.6 [1.6, 1.8]	NA
6000	IsoGraph VAE GPU	GPU	30	11.6 [11.2, 13.1]	1.7 [1.6, 2.0]	0.2 [0.2, 0.4]
6000	WGCNA	CPU	30	35.6 [28.0, 88.5]	2.0 [2.0, 2.8]	NA
12000	IsoGraph VAE	CPU	30	79.7 [52.3, 153.7]	3.0 [2.8, 4.2]	NA
12000	IsoGraph VAE GPU	GPU	30	25.0 [24.3, 33.9]	2.8 [2.7, 4.0]	0.4 [0.4, 0.8]
12000	WGCNA	CPU	30	171.3 [82.9, 246.2]	3.1 [3.1, 3.3]	NA
16000	IsoGraph VAE	CPU	30	226.3 [192.3, 246.8]	6.6 [6.4, 6.7]	NA
16000	IsoGraph VAE GPU	GPU	30	24.6 [22.2, 30.1]	6.2 [5.9, 6.4]	1.2 [1.0, 1.2]
16000	WGCNA	CPU	30	383.5 [365.2, 409.3]	3.7 [3.7, 3.7]	NA

Table S3: Separation of the switch and abundance coordinates, per analysis. One row per analysis (17 rows): genes tested, the median absolute Pearson correlation ($|r|$) between the gene-abundance and isoform-switch coordinates with its first and third quartiles (Q1 and Q3), the fraction of genes with $|r|$ below 0.1, below 0.3, and above 0.5, and the

bioRxiv preprint doi: https://doi.org/10.64898/2026.10.04.756439; this version posted October 11, 2026. The copyright holder for this preprint (which was not certified by peer review) is the author/funder, who has granted bioRxiv a license to display the preprint in perpetuity. It is made available under a CC-BY 4.0 International license.

median signed correlation. The analysis key and region are the identifiers used in the analysis code; the label combines cohort and region. Median $|r|$ ranges from 0.111 in BrainSEQ aging hippocampus to 0.243 in GTEx nucleus accumbens, with 23–46% of genes below 0.1 and 1.1–16.8% above 0.5, over 12,042–13,222 genes per analysis. The quartiles and tail fractions are given because the claim concerns the bulk of the distribution rather than a central value: the two coordinates are largely distinct in every analysis, and close to orthogonal in none. This test requires no deconvolution, so unlike Data S2 it covers all 17 analyses. BrainSEQ aging and BrainSEQ SCZD (schizophrenia) are the BrainSEQ aging and schizophrenia analyses; GTEx, Genotype-Tissue Expression. Backs **Fig. 2b** and **Fig. S12a**.

Analysis key	Cohort	Region	Label	Genes tested	Median $|r|$	Q1 $|r|$	Q3 $|r|$	Fraction $|r| < 0.1$	Fraction $|r| < 0.3$	Fraction $|r| > 0.5$	Median r
brainseq-sczd	BrainSEQ SCZD	caudate	BrainSEQ SCZD: caudate	13222	0.1244	0.0569	0.2362	0.4185	0.8324	0.0439	0.0053
brainseq-aging	BrainSEQ aging	caudate	BrainSEQ aging: caudate	13177	0.1297	0.0597	0.2403	0.4021	0.8274	0.0459	0.0091
brainseq-aging	BrainSEQ aging	hippocampus	BrainSEQ aging: hippocampus	13130	0.111	0.0505	0.1965	0.462	0.9096	0.0113	0.011
brainseq-aging	BrainSEQ aging	dlpfc	BrainSEQ aging: dlpfc	12931	0.1179	0.0537	0.2141	0.4335	0.8633	0.031	0.0148
gtex-aging	GTEx aging	amygdala	GTEx aging: amygdala	12042	0.1918	0.0902	0.3338	0.2755	0.7023	0.0865	0.0088
gtex-aging	GTEx aging	anterior_cingulate_cortex_ba24	GTEx aging: anterior_cingulate_cortex_ba24	12190	0.2031	0.0938	0.3649	0.2661	0.6655	0.1199	0.0175
gtex-aging	GTEx aging	caudate_basal_ganglia	GTEx aging: caudate_basal_ganglia	12424	0.214	0.0974	0.3709	0.2553	0.6533	0.122	0.0076
gtex-aging	GTEx aging	cerebellar_hemisphere	GTEx aging: cerebellar_hemisphere	12416	0.2036	0.0913	0.3602	0.274	0.6678	0.1163	-0.0005
gtex-aging	GTEx aging	cerebellum	GTEx aging: cerebellum	12480	0.1479	0.0694	0.2792	0.3504	0.7788	0.0647	0.007
gtex-aging	GTEx aging	cortex	GTEx aging: cortex	12426	0.1615	0.0719	0.3062	0.3378	0.7416	0.0917	0.0102
gtex-aging	GTEx aging	frontal_cortex_ba9	GTEx aging: frontal_cortex_ba9	12322	0.19	0.0846	0.3484	0.2896	0.6846	0.1109	0.0082
gtex-aging	GTEx aging	hippocampus	GTEx aging: hippocampus	12313	0.1887	0.0898	0.3232	0.2756	0.7172	0.0675	0.0093
gtex-aging	GTEx aging	hypothalamus	GTEx aging: hypothalamus	12648	0.2121	0.1002	0.3683	0.2498	0.6553	0.1184	0.0104

bioRxiv preprint doi: https://doi.org/10.64898/2026.10.04.756439; this version posted October 11, 2026. The copyright holder for this preprint (which was not certified by peer review) is the author/funder, who has granted bioRxiv a license to display the preprint in perpetuity. It is made available under a CC-BY 4.0 International license.

Analysis key	Cohort	Region	Label	Genes tested	Median |*r*|	Q1 |*r*|	Q3 |*r*|	Fraction |*r*| < 0.1	Fraction |*r*| < 0.3	Fraction |*r*| > 0.5	Median *r*
gtex-aging	GTEx aging	nucleus_accumbens_basal_ganglia	GTEx aging: nucleus_accumbens_basal_ganglia	12492	0.2433	0.1118	0.4199	0.2251	0.59	0.1678	0.0067
gtex-aging	GTEx aging	putamen_basal_ganglia	GTEx aging: putamen_basal_ganglia	12100	0.2105	0.0959	0.3721	0.2602	0.6457	0.1198	0.0093
gtex-aging	GTEx aging	spinal_cord_cervical_c_1	GTEx aging: spinal_cord_cervical_c_1	12305	0.1784	0.0831	0.3097	0.2961	0.7352	0.0586	0.0034
gtex-aging	GTEx aging	substantia_nigra	GTEx aging: substantia_nigra	12265	0.1729	0.0807	0.3041	0.304	0.7426	0.0555	0.0072

Table S4: Pooled per-module association rates across the 17 analyses. One row per module set, pooled over all analyses: the number of analyses, the median module count and module size, the mean per-analysis fraction of modules associated with the phenotype, the mean fraction associated on both the switch and abundance coordinates, the mean fraction with an enriched Gene Ontology (GO) term, and the corresponding totals. Methods are IsoGraph (isograph) and weighted gene co-expression network analysis (WGCNA) on gene abundance (wgcna_gene), on switch-only features (wgcna_switch_only), and on the same switch and abundance features (wgcna_multiplex). The comparison is not a like-for-like ranking: the four sets partition the same samples at very different granularities (median 25 modules of 108 genes for IsoGraph against 8 modules of 468 genes for abundance WGCNA), so a per-module rate is computed over different numbers of modules in each row. Backs **Fig. S15**.

Method	Features	Analyses (*n*)	Median modules (*n*)	Median module size	Mean fraction phenotype-associated	Mean fraction both coordinates	Mean fraction GO-enriched	Total phenotype-associated	Total both coordinates
isograph	switch+abundance	17	25	108	0.146	0.052	0.253	54	20
wgcna_gene	abundance	17	8	468	0.178	0.159	0.862	28	24
wgcna_switch_only	switch-only	17	18	159	0.214	0.025	0.184	64	8
wgcna_multiplex	switch+abundance	17	25	242	0.189	0.117	0.649	75	48

Table S5: Constraint and ClinVar density of switch genes and switched exons. One row per module stratum (all switch modules [all], Gene Ontology [GO]-invisible modules [go_invisible], and GO-visible modules [go_visible]) and exon scope (all switch-pair exons [all_exons], or coding exons only [cds]). Columns give the number of annotatable analyses, how many showed switched exons enriched or depleted for ClinVar pathogenic or likely-pathogenic entries relative to other exons of the same transcripts at within-gene permutation $P < 0.05$, the median within-analysis density ratio (switched over background exons, per kilobase [kb]) with its Fisher-combined P, and the median gnomAD loss-of-function observed/expected upper-bound fraction (LOEUF) of switch genes with the Fisher-combined one-sided Mann–Whitney P against the genome-wide reference. Densities count variant call format (VCF) entries, not unique alleles. Backs **Fig. S19a,b**.

Module stratum	Exon scope	Analyses (*n*)	Analyses enriched (*P* < 0.05)	Analyses depleted (*P* < 0.05)	Median density ratio	Median switched-exon density (per kb)	Median background density (per kb)	Density Fisher-combined *P*	Median LOEUF, switch genes	LOEUF Fisher-combined *P*
all	all_exons	10	0	10	0.1601	2.509	16.7	4.51e-17	0.729	1.28e-89

bioRxiv preprint doi: https://doi.org/10.64898/2026.10.04.756439; this version posted October 11, 2026. The copyright holder for this preprint (which was not certified by peer review) is the author/funder, who has granted bioRxiv a license to display the preprint in perpetuity. It is made available under a CC-BY 4.0 International license.

Module stratum	Exon scope	Analyses (*n*)	Analyses enriched ($P < 0.05$)	Analyses depleted ($P < 0.05$)	Median density ratio	Median switched-exon density (per kb)	Median background density (per kb)	Density Fisher-combined *P*	Median LOEUF, switch genes	LOEUF Fisher-combined *P*
go_invisible	all_exons	7	0	6	0.1618	3.321	15.63	8.17e-11	0.765	2.31e-54
go_visible	all_exons	9	0	8	0.1619	2.383	12.71	5.67e-14	0.7145	1.24e-64
all	cds	10	0	10	0.1822	2.965	17.11	5.83e-16	0.729	1.28e-89
go_invisible	cds	7	0	7	0.1808	3.921	16.16	1.29e-10	0.765	2.31e-54
go_visible	cds	9	0	8	0.1858	2.74	13.46	4.04e-14	0.7145	1.24e-64

Table S6: Splicing-QTL specificity contrast, all analyses. One row per network method and module set: the number of analyses pooled, the fixed-effect inverse-variance pooled ratio of the splicing quantitative trait locus (sQTL) odds ratio to the expression QTL (eQTL) odds ratio for module membership (paired within analysis, which removes the shared *cis*-QTL depletion of network genes), its 95% confidence interval (CI) and *P* value, and between-analysis heterogeneity (I^2). Methods are IsoGraph (isograph) and classical weighted gene co-expression network analysis (WGCNA) on the same switch-only (wgcna_switch_only) or switch-plus-abundance (wgcna_multiplex) features. Module sets are all modules, phenotype-associated modules (pheno_sig_modules), and Gene Ontology (GO)-invisible and GO-visible phenotype-associated modules. Backs **Fig. S23b,c**.

Method	Module set	Analyses (*n*)	sQTL/eQTL odds-ratio ratio	95% CI, lower	95% CI, upper	*P*	I^2
isograph	all_modules	17	1.033	1.006	1.062	0.0179	0.6036
isograph	pheno_sig_modules	10	1.065	1.01	1.123	0.0198	0.104
wgcna_switch_only	pheno_sig_modules	6	0.9273	0.856	1.005	0.0646	0.4648
wgcna_multiplex	pheno_sig_modules	8	1.084	1.025	1.146	0.00444	0.6386
isograph	go_invisible_modules	7	1.08	0.9873	1.182	0.0927	0
wgcna_switch_only	go_invisible_modules	6	0.9292	0.8567	1.008	0.0762	0.4589
wgcna_multiplex	go_invisible_modules	4	1.006	0.9121	1.109	0.905	0.6106
isograph	go_visible_modules	9	1.049	0.9866	1.116	0.126	0.3384
wgcna_switch_only	go_visible_modules	2	0.9694	0.7851	1.197	0.773	0
wgcna_multiplex	go_visible_modules	8	1.095	1.036	1.157	0.00126	0.5034

Table S7: Splicing-QTL specificity contrast on the analyses shared by all methods. As Table S6, restricted to the analyses in which IsoGraph and both matched weighted gene co-expression network analysis (WGCNA) comparators produced the relevant module set, so that the three methods are compared on identical inputs. Columns give the number of analyses pooled, the fixed-effect pooled ratio of the splicing quantitative trait locus (sQTL) odds ratio to the expression QTL (eQTL) odds ratio for module membership, its 95% confidence interval (CI) and *P* value, and between-analysis heterogeneity (I^2). Method and module-set labels are as in Table S6. Backs **Fig. S23c**.

Method	Module set	Analyses (*n*)	sQTL/eQTL odds-ratio ratio	95% CI, lower	95% CI, upper	*P*	I^2
isograph	all_modules	5	1.03	0.9814	1.082	0.228	0.5867
isograph	pheno_sig_modules	5	1.04	0.9793	1.105	0.2	0
wgcna_switch_only	pheno_sig_modules	5	0.9302	0.8528	1.014	0.102	0.5704

bioRxiv preprint doi: https://doi.org/10.64898/2026.10.04.756439; this version posted October 11, 2026. The copyright holder for this preprint (which was not certified by peer review) is the author/funder, who has granted bioRxiv a license to display the preprint in perpetuity. It is made available under a CC-BY 4.0 International license.

Method	Module set	Analyses (*n*)	sQTL/eQTL odds-ratio ratio	95% CI, lower	95% CI, upper	*P*	I^2
wgcna_multiplex	pheno_sig_modules	5	1.059	0.9977	1.124	0.0597	0.6318
isograph	go_invisible_modules	4	1.072	0.9483	1.211	0.267	0
wgcna_switch_only	go_invisible_modules	5	0.9293	0.8518	1.014	0.0989	0.5671
wgcna_multiplex	go_invisible_modules	3	0.9955	0.9021	1.099	0.929	0.5113
isograph	go_visible_modules	5	1.025	0.9604	1.095	0.452	0
wgcna_switch_only	go_visible_modules	1	0.999	0.6682	1.494	0.996	0
wgcna_multiplex	go_visible_modules	5	1.076	1.014	1.141	0.0147	0.5007

Table S8: Module-level *cis* control of isoform choice. One row per age-selected IsoGraph module with a fitted gene in the BrainSEQ within-donor allelic test. Columns give the module's age trait (linear or spline age model) and effect, the genes fitted, the genes with a transcript pair at Benjamini–Hochberg $q < 0.05$ and their fraction, the module's age-association rank within its trait (0 weakest, 1 strongest), and the number of modules in that trait. Dorsolateral prefrontal cortex (dlpfc) ranks are missing because it has too few modules per trait to rank. Backs **Fig. S21e**.

Region	Module	Age trait	Age effect	Genes fitted	Genes with pair at *q* < 0.05	Fraction	Age rank within trait	Modules in trait (*n*)
caudate	M000	Age_linear	0.2623	84	29	0.3452	1	3
caudate	M002	Age_linear	0.1548	92	34	0.3696	0.5	3
caudate	M004	Age_linear	0.1546	75	30	0.4	0	3
caudate	M003	Age_spline	2.941	100	30	0.3	1	4
caudate	M006	Age_spline	0.8375	80	40	0.5	0	4
caudate	M008	Age_spline	1.672	38	17	0.4474	0.6667	4
caudate	M011	Age_spline	1.422	12	5	0.4167	0.3333	4
dlpfc	M000	Age_spline	-2.046	58	20	0.3448		
dlpfc	M007	Age_spline	-1.507	32	11	0.3438		
dlpfc	M008	Age_linear	0.2285	25	6	0.24		
hippocampus	M000	Age_linear	0.2664	28	12	0.4286	0.1538	14
hippocampus	M001	Age_linear	0.4398	44	16	0.3636	0.9231	14
hippocampus	M002	Age_linear	0.3454	28	6	0.2143	0.4615	14
hippocampus	M003	Age_linear	0.4071	13	2	0.1538	0.7692	14
hippocampus	M004	Age_linear	0.4236	77	22	0.2857	0.8462	14
hippocampus	M006	Age_linear	0.1738	7	1	0.1429	0	14
hippocampus	M007	Age_linear	0.3472	31	6	0.1935	0.5385	14
hippocampus	M008	Age_linear	0.3936	22	9	0.4091	0.6923	14
hippocampus	M009	Age_linear	0.3035	12	5	0.4167	0.3846	14
hippocampus	M010	Age_linear	0.2809	3	3	1	0.2308	14
hippocampus	M011	Age_linear	0.3874	8	6	0.75	0.6154	14
hippocampus	M012	Age_linear	0.2845	4	0	0	0.3077	14
hippocampus	M013	Age_linear	0.2474	1	0	0	0.0769	14
hippocampus	M015	Age_linear	0.4505	1	1	1	1	14

bioRxiv preprint doi: https://doi.org/10.64898/2026.10.04.756439; this version posted October 11, 2026. The copyright holder for this preprint (which was not certified by peer review) is the author/funder, who has granted bioRxiv a license to display the preprint in perpetuity. It is made available under a CC-BY 4.0 International license.

Table S9: Synthetic benchmark scenarios. One row per scenario of the synthetic grid. Swept parameters were crossed within a scenario; the number of datasets is the number of grid cells times seeds per cell (30 for idealized switching, 15 for the scale scenarios, and 20 otherwise), and each method was run once per dataset. Scenario and parameter names are the identifiers used in the committed grid configuration, whose values are listed here.

Scenario	Genes (*n*)	Samples (*n*)	Swept parameters	Fixed parameters	Grid cells	Seeds per cell	Datasets
idealized_switching	400	160	switching_fraction = 0.1, 0.25, 0.5, 0.75; noise_sd = 0.1, 0.25, 0.4		12	30	360
unequal_isoform_abundance	400	160	abundance_imbalance = 1, 4, 10, 25	switching_fraction = 0.25; noise_sd = 0.2	4	20	80
non_switching_background	400	160	switching_fraction = 0.05, 0.1, 0.25, 0.5	noise_sd = 0.25	4	20	80
noise_stress	400	160	count_dispersion = 3, 7, 15, 30; noise_sd = 0.05, 0.1, 0.25, 0.5	switching_fraction = 0.25	16	20	320
feature_space_interactions	400	160	interaction_strength = 0.0, 0.5, 1.0, 2.0, 3.0; interaction_fraction = 0.25, 0.5, 0.75	switching_fraction = 0.25; noise_sd = 0.25	15	20	300
abundance_switch_mixed	200; 500	100; 200	abundance_fraction = 0.0, 0.3, 0.5, 0.7, 1.0	switching_fraction = 0.25; noise_sd = 0.25	20	20	400
negative_control_noise	400	160	noise_sd = 0.5, 1.0	switching_fraction = 0.0	2	20	40
rna_degradation	400	160	degradation_3p_bias = 0.0, 0.5, 1.0, 2.0	switching_fraction = 0.25; noise_sd = 0.25	4	20	80
rna_degradation_coupled	400	160	degradation_3p_bias = 0.0, 0.5, 1.0, 2.0	switching_fraction = 0.25; abundance_fraction = 0.4; dual_signal_fraction = 0.5; noise_sd = 0.25	4	20	80
multi_isoform_switch	400	160	noise_sd = 0.1, 0.25	switching_fraction = 0.25; n_transcripts_per_gene = 4	2	20	40
cell_composition	400	160	cell_composition_cv = 0.0, 0.1, 0.2, 0.3	switching_fraction = 0.25; noise_sd = 0.25	4	20	80
batch_effects	400	160	batch_effect_sd = 0.0, 0.25, 0.5, 1.0	switching_fraction = 0.25; noise_sd = 0.25	4	20	80
library_depth	400	160	library_depth_cv = 0.25, 0.5, 0.75, 1.0	switching_fraction = 0.25; noise_sd = 0.25	4	20	80
genetic_anchoring	400	200	noise_sd = 0.25, 0.4; genetic_effect = 0.5, 1.0, 2.0	switching_fraction = 0.25; genetic_module_fraction = 1.0; genetic_maf = 0.3	6	20	120
scale	1000; 3000; 6000; 12000	240	switching_fraction = 0.15, 0.25	noise_sd = 0.25	8	15	120

bioRxiv preprint doi: https://doi.org/10.64898/2026.10.04.756439; this version posted October 11, 2026. The copyright holder for this preprint (which was not certified by peer review) is the author/funder, who has granted bioRxiv a license to display the preprint in perpetuity. It is made available under a CC-BY 4.0 International license.

**Scenario**	**Genes (*n*)**	**Samples (*n*)**	**Swept parameters**	**Fixed parameters**	**Grid cells**	**Seeds per cell**	**Datasets**
scale_realistic	16000	300	switching_fraction = 0.15, 0.25	noise_sd = 0.25	2	15	30

bioRxiv preprint doi: https://doi.org/10.64898/2026.10.04.756439; this version posted October 11, 2026. The copyright holder for this preprint (which was not certified by peer review) is the author/funder, who has granted bioRxiv a license to display the preprint in perpetuity. It is made available under a CC-BY 4.0 International license.

Supplementary Data

Data S1. Discovery cohorts. One row per analysis (17 analyses over 16 region-level cohorts; the BrainSEQ schizophrenia caudate analysis includes the 238 control donors of the aging caudate analysis). Each donor contributes one sample per analysis. Age is in years; Genotype-Tissue Expression (GTEx) exact age is released top-coded at 70. BrainSEQ reports postmortem interval (PMI, h) and GTEx tissue ischemic time (min). Genes and transcripts are counts after the bundle expression filter, before the prevalence filter applied at module fitting. The CSV adds library kit, self-reported ancestry, manner of death or Hardy scale, feature counts before filtering, and the single-nucleus RNA-sequencing (snRNA-seq) reference used for MuSiC deconvolution.

Data S2. Switch-unique genes before and after adjustment for estimated cellular composition. One row per analysis for the 12 analyses with an anatomically matched single-nucleus reference: genes tested, switch-unique genes before and after adding the estimated cell-type proportions to the conditional model, the number of unadjusted genes that remain (`n_overlap`) and the number that adjustment newly introduces (`n_new`), genes significant on both the switch and abundance coordinates in each arm, the number of cell types and samples covered by the deconvolution, and the number of module-by-cell-type marker tests with the counts reaching false discovery rate (FDR) < 0.05 for depletion and for enrichment. Because adjustment both removes and adds genes, the adjusted set is not nested in the unadjusted one, so `n_overlap` divided by the unadjusted count – not the ratio of the two counts – is the persistence statistic. The five Genotype-Tissue Expression (GTEx) regions with no matched reference are absent by design rather than entered as zeros. Backs **Fig. 2c,d** and **Fig. S14d**.

Data S3. Per-region module trust funnel. One row per cohort, region and method for the six anatomically paired cohort-region analyses. Columns cover split-half stability (module count, trusted count, and fraction above a size-matched permutation null at false discovery rate [FDR] < 0.05, with the median co-assignment density and its null), switch-driver reproducibility (median shared-gene driver-loading Spearman correlation and the fraction of positive pairs; IsoGraph only, as classical weighted gene co-expression network analysis [WGCNA] has no transcript-level drivers), the split-half aging counts (pairs tested, pairs significant for age in both halves, and pairs agreeing in sign), and complementarity to the abundance baseline. Best-match Jaccard is reported for description only: it is confounded by partition granularity, which is why the trust criterion is the chance-calibrated co-assignment test. Backs **Fig. 3a–d**.

Data S4. Per-analysis module association rates for all four module sets. The per-region ledger behind Table S4: one row per cohort, region, and method (68 rows) giving the module count, median module size, Gene Ontology enrichment count and fraction, and the counts and fractions of modules associated with the phenotype and with both coordinates. It is included because the pooled means hide the fact that the two cohorts order the methods differently, which is why the pooled ordering is not read as a method ranking. Backs **Fig. S15**.

Data S5. Split-half module ledger. One row per module per method (190 rows): module size, co-assignment density, the mean of its size-matched permutation null, permutation *P*, Benjamini–Hochberg *q*, the trusted flag and the best-match Jaccard against the most-overlapping half-fit module. Every module behind the 93 of 118 and 62 of 72 trusted counts is listed, so the counts can be audited module by module. Backs **Fig. 3b**.

Data S6. Split-half agreement across the Leiden resolution sweep. Mean adjusted Rand index (ARI) and normalized mutual information between the two halves of each donor split, over five seeds,

bioRxiv preprint doi: https://doi.org/10.64898/2026.10.04.756439; this version posted October 11, 2026. The copyright holder for this preprint (which was not certified by peer review) is the author/funder, who has granted bioRxiv a license to display the preprint in perpetuity. It is made available under a CC-BY 4.0 International license.

at eight Leiden resolutions (0.5, 1, 2, 3, 5, 8, 12, and 20) for each cohort and region. The production resolution of 2.0 is flagged in `is_production`. The weighted gene co-expression network analysis (WGCNA) rows carry no resolution, because there is none to set, and are kept so the sweep is read against the fixed baseline rather than against itself. Averaged across regions, agreement varies little over the whole sweep (mean ARI 0.37–0.47, production 0.42), and the best resolution differs by region (0.5, 0.5, 1, 3, 20 and 20), so no resolution is uniformly best and the production value is not a tuned optimum. Per-region production values span ARI 0.19–0.57 for IsoGraph against 0.29–0.71 for abundance WGCNA on the same splits, the same granularity contrast the funnel reports throughout.

Data S7. Cross-cohort eigengene projection ledger. One row per trusted module per transfer direction (155 rows): the frozen-weight signed eigengene correlation against its feature-type-matched null with Benjamini–Hochberg *q*, the module's age correlation in each cohort, that correlation expressed as a *z* statistic against same-weight random projections, and whether the two cohorts agree in sign. The final columns give the same sign agreement computed on the raw projected age correlations. These are the reason the reported statistic is standardized: among the modules significant in both cohorts on raw correlations, the three BrainSEQ-to-Genotype-Tissue Expression (GTEx) region pairs give sign agreement of 8 of 8 (caudate), 8 of 8 (dorsolateral prefrontal cortex to frontal cortex BA9) and 0 of 13 (hippocampus) – two regions unanimous in one direction and one unanimous in the other, which is a property of each target cohort's own age-correlated structure rather than of the modules (**Data S27**). Backs **Fig. 3e**.

Data S8. Cross-cohort matched-pair count against its permutation nulls. The full grid of two methods by three statistics (covariate-free Pearson correlation, covariate-adjusted linear age term, covariate-adjusted natural cubic spline block *F* test) by three covariate modes by two nulls. The `age` null regenerates the target-cohort age association under the age-null while holding module construction and matching fixed; the `matching` null holds every age statistic fixed and permutes which target module each source module is matched to, and is the stricter of the two. The `is_published_statistic` flag marks the covariate-free Pearson arm quoted in the main text. Against the matching null that arm gives 1 of 38 ($P = 0.91$) for IsoGraph and 2 of 52 ($P = 0.15$) for weighted gene co-expression network analysis (WGCNA). The spline sensitivity arm with the matching null and `complement` covariate mode gives 0 of 38 ($P = 1$) and 5 of 52 ($P = 0.16$). The `complement` mode adds covariates outside the discovery residualization set; `full` adds the complete covariate set and `none` adds none. Spline significance uses a three-degree-of-freedom block *F* test, with direction taken from the corresponding linear age coefficient. Neither method exceeds the matching null in the reported Pearson or spline arm.

Data S9. Functional preservation of matched cross-cohort module pairs. Matched BrainSEQ–Genotype-Tissue Expression (GTEx) module pairs compared with a null that re-pairs each module with a random target module drawn from the same gene-count decile, because all three similarity measures increase with module size. Median gene Jaccard across matched pairs is 0.04, the gene-level overlap these measures are asked to look past. Rows with `n_finite` of zero are untested rather than negative: transcript-structure similarity had no computable pair in any region, and cell-type composition profiles are produced only for the IsoGraph arm. Both the linear and the spline age model are included; the main text reports the linear arm. Backs **Fig. 3f**.

Data S10. satuRn concordance of IsoGraph switch genes, per analysis. One row per cohort, region, and trait (17 analyses). Columns give the number of satuRn-tested genes, of switch genes (members of trait-associated IsoGraph switch modules), and of background genes, and the median satuRn differential transcript usage evidence ($-\log_{10}$ minimum isoform *P*) in each set. The primary test is the one-sided Mann–Whitney *P* with its rank-biserial effect size. The secondary is the odds ratio of switch-module membership per unit of evidence, adjusted for transcript count, with its *P*. Analyses

bioRxiv preprint doi: https://doi.org/10.64898/2026.10.04.756439; this version posted October 11, 2026. The copyright holder for this preprint (which was not certified by peer review) is the author/funder, who has granted bioRxiv a license to display the preprint in perpetuity. It is made available under a CC-BY 4.0 International license.

with no switch genes are kept with empty statistics because nothing could be tested; they are not null results. Backs **Fig. S17**.

Data S11. Gene-level corroboration of the switch layer by junction usage. One row per analysis and switch set. Switch-positive genes are members of a trait-associated IsoGraph module (module set) or genes whose own switch coordinate is trait-associated (marginal set; BrainSEQ caudate only). Percent-spliced-in (PSI)-positive genes have at least one junction-level event (a PSI event in BrainSEQ, or a within-gene junction-usage feature in Genotype-Tissue Expression [GTEx]) associated with the trait under the same covariate model (within-gene Bonferroni, then Benjamini–Hochberg across genes, false discovery rate [FDR] ≤ 0.05). The adjusted odds ratio is from a logistic model of PSI status on switch status and log transcript count. The CSV adds the Fisher exact test, the PSI-positive rates in switch and background genes, and, for BrainSEQ, the junction-level resolution counts. Analyses with no switch-positive genes are listed with empty statistics.

Data S12. Module context and held-out DTU evidence, per analysis. One row per anatomically paired cohort-region analysis (10 split directions each). Columns give the tested genes and their coverage of the satuRn-tested universe, and the partial correlation of module context with held-out differential transcript usage (DTU) evidence (mean, median, range, and positive directions). Each correlation is given with its stratified permutation *P* and Benjamini–Hochberg *q*, before and after conditioning on the co-expression context, followed by the co-expression context's own correlation and the resulting pattern. The sub-threshold columns give the fraction reaching held-out nominal Simes $P < 0.05$ among genes with discovery Benjamini–Hochberg $q \geq 0.05$, in the top and bottom tertiles of module context, and the median adjusted odds ratio across split directions. These descriptive odds ratios adjust for discovery evidence and transcript features, without the additional co-expression predictor; nominal held-out support is not a false discovery rate (FDR)-significant replication call. The final columns are a post hoc check that recomputes the statistic without modules of 900 or more genes and without each split's largest module. The decision rule recorded in the analysis code ($q < 0.05$ in at least four of six analyses, including one per cohort) applied to the model before co-expression adjustment and was met by four analyses. Three remained significant after co-expression adjustment; this additional model did not meet the four-of-six criterion. Backs **Fig. S18**.

Data S13. RNA-binding-protein motif regulons of the switch modules. One row per analysis, module, and RNA-binding protein (RBP; 11,040 cells). Columns give the module size, Gene Ontology (GO) visibility, and switch-gene counts; the hypergeometric test of RBP site-switch over-representation (expected count, enrichment, *P*, and Benjamini–Hochberg *q*); the opportunity-adjusted binomial model (coefficient, standard error, odds ratio with 95% confidence interval [CI], *P*, *q*, and fit status); and whether the cell is supported by both tests. Backs **Fig. S20a,b**.

Data S14. ENCODE eCLIP binding at switched and constitutive exons. One row per testable RNA-binding protein (RBP; 35). Columns give the nominated genes; the fraction with an enhanced crosslinking and immunoprecipitation (eCLIP) peak over switched and over constitutive exon intervals and their difference; the discordant gene counts, exact McNemar *P*, matched odds ratio with 95% confidence interval (CI), and false discovery rate (FDR); peak density per kilobase in each interval class with a gene-level bootstrap CI of their ratio; and whether binding is preferential and supported. Backs **Fig. S20c**.

Data S15. Within-donor allelic test of isoform choice, per region. One row per BrainSEQ region for the Benjamini–Hochberg-tested set of switch pairs. Columns give the pairs tested and fitted, genes fitted, pairs at nominal $P < 0.05$, and pairs and genes at Benjamini–Hochberg $q < 0.05$; the sign agreement of the within-donor effect with the between-donor dosage association (all fitted pairs and $q < 0.05$ pairs); fits that did not converge or reached the $|\beta| = 10$ bound; the homozygous-at-lead null

bioRxiv preprint doi: https://doi.org/10.64898/2026.10.04.756439; this version posted October 11, 2026. The copyright holder for this preprint (which was not certified by peer review) is the author/funder, who has granted bioRxiv a license to display the preprint in perpetuity. It is made available under a CC-BY 4.0 International license.

(fits, fraction at $P < 0.05$, and the genomic inflation factor λ_{GC}) and λ_{GC} of the heterozygous test; the Spearman correlation of the mixed-model effect with the score-test Z; and the median lead-to-heterozygous-site distance. The module-axis columns test whether, within a gene, the effect tracks each pair's module polarity, against a within-gene permutation null. The module-*cis* columns give the correlation of module *cis*-controlled-gene rate with age-association rank and its permutation *P*; the final row pools caudate and hippocampus. The last two columns count the separately fitted colocalization-nominated pairs, which carry a *q* only where they are also in the tested set. Backs **Fig. 5a** and **Fig. S21**.

Data S16. sQTL colocalization nominations by signal-level and regional fallback support. Every analysis-by-gene combination with a splicing quantitative trait locus (sQTL) posterior probability of a shared causal variant (PP4) ≥ 0.8 in the all-introns colocalization (127 rows). The aging and schizophrenia (SCZD) gene sets overlap, so the rows collapse to 119 gene-by-trait cells in 116 genes. Columns give the module and Gene Ontology (GO) visibility, the sQTL and expression QTL (eQTL) PP4, whether the call is sQTL-preferential (eQTL PP4 < 0.5), the tissues tested and colocalizing on each modality, and the headline tissue, intron, estimator (`coloc.susie` or the `coloc.abf` fallback), and prior robustness. The headline PP4 is a maximum over correlated tissues and should be read with the tissue count. The sQTL-preferential flag is a selection on the sQTL call, not an estimate of splicing specificity; Data S17 is the unbiased comparison. Of the 119 cells, 33 were scored by `coloc.susie` in at least one tissue; the remaining 86 rest on the `coloc.abf` fallback.

Data S17. Paired sQTL and eQTL colocalization contrast. One row per analysis and a pooled row. Columns give the genes tested; the genes colocalizing with splicing quantitative trait loci (sQTLs), expression QTLs (eQTLs), both, only one modality, or neither (posterior probability of a shared causal variant [PP4] ≥ 0.8); the McNemar *P* of the discordant counts and the splicing share among them; and paired Wilcoxon comparisons of the conditional and raw PP4 of the two modalities.

Data S18. BrainSEQ switch- and abundance-axis colocalization contrast. One row per analysis and a pooled row, in European-ancestry BrainSEQ donors with in-sample linkage disequilibrium (LD). Columns give the genes testable on both IsoGraph axes; the genes colocalizing (posterior probability of a shared causal variant [PP4] ≥ 0.8) on the switch axis, the abundance axis, both, one axis only, or neither; the exact McNemar *P* and the switch share of the discordant genes; and paired Wilcoxon comparisons of the conditional and raw PP4. Backs **Fig. S22a**.

Data S19. Colocalization events and their mapping to IsoGraph switch pairs. One row per colocalization event, defined as a gene-by-trait nomination in one tissue with a splicing quantitative trait locus (sQTL) posterior probability of a shared causal variant (PP4) ≥ 0.8 (410 events over the 119 nominations of Data S16). Rows are sorted with the 115 events whose colocalizing junction is carried by an isoform of the tissue-matched switch pair first; these events cover 56 nominations, 21 of them signal-level. Columns give the analysis, trait, gene, and tissue; whether the nomination is signal-level (`coloc.susie` in at least one tissue); the estimator, sQTL and expression QTL (eQTL) PP4, prior robustness, and fallback reason of the event; the intron and junction; the junction-carrying transcripts and Gene Ontology (GO) visibility; whether the junction is carried by a switch-pair transcript, the switch pairs (up to 15 per gene) and the correlation of junction usage with switch polarity; the matched BrainSEQ region and whether the junction-carrying transcript also maps to a switch pair there; and whether the posterior-overlap layer (Data S21) also resolves the same gene-by-trait cell to a switch pair. `coloc.abf` rows name the representative intron of the Genotype-Tissue Expression (GTEx) release only. Risk-allele direction is resolved only in the posterior-overlap layer. Cross-cohort pair mapping does not establish replication of the phenotype association. Locus notes: *TMEM175* (amyotrophic lateral sclerosis [ALS], PP4 = 0.998) lies in a gene-dense region that also colocalized with Parkinson's and Alzheimer's disease at different lead variants, and few of its probes passed summary-

bioRxiv preprint doi: https://doi.org/10.64898/2026.10.04.756439; this version posted October 11, 2026. The copyright holder for this preprint (which was not certified by peer review) is the author/funder, who has granted bioRxiv a license to display the preprint in perpetuity. It is made available under a CC-BY 4.0 International license.

data-based Mendelian randomization (SMR) without rejection by the heterogeneity in dependent instruments (HEIDI) test (Data S20); *SNCA* (dementia with Lewy bodies, PP4 = 0.970) maps to a switch pair in hypothalamus and cerebellum but not in cortex; *C9orf72* (ALS) colocalized on splicing in eight brain tissues (sQTL PP4 0.83–0.99, eQTL PP4 $< 10^{-8}$) through `coloc.abf` only, maps to a cerebellar switch pair and also colocalized on the BrainSEQ switch axis (dorsolateral prefrontal cortex PP4 = 0.98; Data S18), and because the ALS association there tags the repeat-expansion haplotype it is read as a haplotype-linked splicing effect. Backs **Fig. 5b,c**.

Data S20. SMR and HEIDI for the colocalization nominations. One row per primary confirmatory probe, one per gene and quantitative trait locus (QTL) class (410 splicing QTL [sQTL] and 410 expression QTL [eQTL] probes). Columns give the analysis, trait, gene, QTL class, probe, and tissue; the top *cis*-QTL variant; the colocalization posterior probability of a shared causal variant (PP4) and estimator for the probe; the summary-data-based Mendelian randomization (SMR) estimate, standard error, and *P* with its Bonferroni threshold; the heterogeneity in dependent instruments (HEIDI) test *P* and number of variants; the instrument *F* statistic and weak-instrument flag; and the SMR status and its agreement with colocalization. “No instrument” means untested, not negative. Backs **Fig. S22b**.

Data S21. Posterior-overlap-nominated isoform events and their mapping to IsoGraph switch pairs. One row per overlap-supported quantitative trait locus (QTL) event (467 events), sorted with the 76 events whose junction maps into a tissue-matched switch pair first. Columns give the analysis, trait, and case; the gene, QTL type, and tissue; the lead variant, its risk allele, and the risk allele’s QTL effect; the junction and its credible-set posterior-overlap score (CLPP); Gene Ontology (GO) visibility; whether the junction is carried by a switch-pair transcript, the switch pairs (up to 15 per gene) and the junction-carrying transcripts; the correlation of junction usage with the switch polarity; and the matched BrainSEQ region with whether the junction-carrying transcript also maps to a switch pair there. This is cross-cohort pair mapping, not replication of the phenotype association. Corroborates the colocalization events of Data S19.

Data S22. Partitioned heritability of the switch-derived QTL annotations. Stratified linkage disequilibrium (LD) score regression of five genome-wide association study (GWAS) traits on variants that are Genotype-Tissue Expression (GTEx) brain quantitative trait loci (QTLs) of the aging switch genes (splicing QTL [sQTL], expression QTL [eQTL], or either) and, for schizophrenia, of the schizophrenia (SCZD) switch genes, conditional on baselineLD v2.2. Single-annotation models test each annotation alone; joint models include the sQTL and eQTL annotations together. Columns give the proportion of variants and of heritability, the enrichment and its *P*, and the per-variant coefficient with its standard error, *Z* and one-sided *P*. `coef_q_bh` is the Benjamini–Hochberg *q* across the 15 single-annotation tests of the aging switch genes; joint-model and SCZD-module rows are descriptive and have no *q*. Backs **Fig. 5e**.

Data S23. Module concentration of posterior-overlap-supported genes. One row per trait and aging cohort. Columns give the overlap-score-tested pool, the supported genes and the modules containing them, the observed concentration (sum of squared per-module counts) with its null mean and empirical *P*, and the fraction of overlap-supported genes in MAGMA-anchored modules (modules with a competitive gene-set $P < 0.05$) with the hypergeometric *P* under the overlap-score-tested pool and under all module genes. Dementia with Lewy bodies (LBD) in BrainSEQ had no overlap-supported genes. Backs **Fig. S24**.

Data S24. MAGMA competitive gene-set tests of every module. One row per module, trait and pipeline (8,064 rows): IsoGraph modules at Leiden resolution 2.0 (production) and 5.0 (sensitivity) and classical abundance-only weighted gene co-expression network analysis (WGCNA) modules, tested against eight genome-wide association studies (GWAS; schizophrenia, bipolar disorder, major

bioRxiv preprint doi: https://doi.org/10.64898/2026.10.04.756439; this version posted October 11, 2026. The copyright holder for this preprint (which was not certified by peer review) is the author/funder, who has granted bioRxiv a license to display the preprint in perpetuity. It is made available under a CC-BY 4.0 International license.

depressive disorder, Alzheimer's disease, Parkinson's disease, dementia with Lewy bodies, amyotrophic lateral sclerosis, and stroke). Columns give the cohort, region and module identifier, module size, whether the module has at least 900 genes, the MAGMA coefficient, its standardized form and standard error, the *P* value, and the Benjamini–Hochberg false discovery rate (FDR) within trait and pipeline. Module identifiers are specific to the fit that produced them. Backs **Fig. S25**.

Data S25. Long-read transcript detection and reciprocal usage of colocalization-anchored pairs. One row per gene (55) whose colocalizing junction is carried by an isoform of a tissue-matched switch pair. Columns give the traits and the maximum splicing quantitative trait locus (sQTL) posterior probability of a shared causal variant (PP4); the distinct transcript pairs, those detected in the long-read data, and those that are switch-like, overall and restricted to pairs with an anchored isoform whose mean within-gene fraction is at least 0.05 (the well-expressed subset). Distinct unordered pairs are the primary unit. The table also gives counts over anchored–partner orientations, in which a pair anchored from both transcripts appears twice (sensitivity analysis); the strongest usage correlation; the anchored isoform's maximum mean fraction; and whether the gene has at least one switch-like pair overall and at that abundance (the source "confirmed" fields). These calls do not test age association. Backs **Fig. 6a,b**.

Data S26. Short-read junction corroboration of colocalization-prioritized transcript pairs. One row per nominated transcript pair and primary BrainSEQ region (260 rows) for the 55 genes whose signal-level splicing quantitative trait locus (sQTL) colocalization junction is carried by an isoform of a tissue-matched IsoGraph switch pair. Columns give the gene, trait(s), Genotype-Tissue Expression (GTEx) tissue of the colocalization and its maximum sQTL posterior probability of a shared causal variant (PP4); the implicated junction and the two versioned transcripts of the pair; the BrainSEQ region and its anatomical match class (exact or generic-cortex proxy); whether the implicated junction itself discriminates the two alternatives and whether the recount model is two-sided; the number of nominating events; the coverage thresholds; the donors with completed recounts, the covered donors (at least 10 pair-discriminating fragments), the donors with at least one fragment for each alternative, and their fraction; the median total fragments and minor-form usage; the measurement status (measured, pair not counted, not two-sided, or insufficient coverage); and the descriptive donor-level Spearman concordance between junction-based and transcript-quantification usage, raw and covariate-adjusted, with its status. Counts pool haplotypes, including unassigned fragments, over adult control donors. Unmeasured rows are untestable rather than negative, and the concordance correlations share reads with the transcript estimates and carry no *P* values. Backs **Fig. 6c**.

Data S27. Raw against null-standardized projected-age sign agreement, per region. One row per method, transfer direction and region pair, with the switch-axis sign convention appended to the IsoGraph rows. The two arms are counted over different denominators and both are given: raw sign agreement over the modules whose projected age correlation is significant in both cohorts, and standardized sign agreement over every age-testable module. The raw arm is the reason the reported statistic is standardized – it is near-unanimous within a region and can be unanimous in either direction (8 of 8, 8 of 8, and 0 of 13 for the three BrainSEQ-to-Genotype-Tissue Expression [GTEx] pairs), which tracks the target cohort's own age-correlated structure rather than the modules. The orientation columns give the second reason: the switch axis of a gene is fitted independently in each cohort, and about 45% of the genes that can be oriented (2,505–2,616 of 5,450–5,739 per region) carry the opposite sign in the two cohorts, so a raw projected coefficient inherits a sign the projection did not earn. These columns are empty for weighted gene co-expression network analysis (WGCNA), which projects abundance features and has no switch axis to orient.

Data S28. Short-read junction-usage support for posterior-overlap-anchored pairs. One row per candidate BrainSEQ percent-spliced-in (PSI) event (173 rows) for the 30 genes whose posterior-

bioRxiv preprint doi: https://doi.org/10.64898/2026.10.04.756439; this version posted October 11, 2026. The copyright holder for this preprint (which was not certified by peer review) is the author/funder, who has granted bioRxiv a license to display the preprint in perpetuity. It is made available under a CC-BY 4.0 International license.

overlap-supported junction is carried by an isoform of the tissue-matched IsoGraph switch pair. Columns give the gene, trait, Genotype-Tissue Expression (GTEx) tissue of the colocalization, and the BrainSEQ region tested with its anatomical match class; the anchored junction, its credible-set posterior-overlap score (CLPP), and risk allele; the event and its type; the number of samples; the median PSI and minor-form usage (min(median PSI, 1 − median PSI)); the fraction of samples with minor-form usage of at least 0.05; and the verdict. A positive usage verdict denotes detectable alternative usage at the specified threshold, not phenotype replication. Events in GTEx tissues without a BrainSEQ counterpart are untestable rather than negative.
